# Safety and Efficacy of Intermediate- and Therapeutic-Dose Anticoagulation for Hospitalised Patients with COVID-19: A Systematic Review and Meta-Analysis

**DOI:** 10.3390/jcm11010057

**Published:** 2021-12-23

**Authors:** Stefanie Reis, Maria Popp, Benedikt Schmid, Miriam Stegemann, Maria-Inti Metzendorf, Peter Kranke, Patrick Meybohm, Stephanie Weibel

**Affiliations:** 1 Department of Anaesthesiology, Intensive Care, Emergency and Pain Medicine, University Hospital Wuerzburg, 97080 Wuerzburg, Germany; Reis_S@ukw.de (S.R.); Popp_M4@ukw.de (M.P.); Schmid_B@ukw.de (B.S.); Kranke_P@ukw.de (P.K.); Meybohm_P@ukw.de (P.M.); 2 Department of Infectious Diseases and Respiratory Medicine, Charité–Universitätsmedizin Berlin, Corporate Member of Freie Universität Berlin and Humboldt—Universität zu Berlin, 10117 Berlin, Germany; miriam.stegemann@charite.de; 3 Cochrane Metabolic and Endocrine Disorders Group, Institute of General Practice, Medical Faculty of the Heinrich-Heine—University Düsseldorf, 40225 Düsseldorf, Germany; maria-inti.metzendorf@med.uni-duesseldorf.de

**Keywords:** anticoagulant therapy, coronavirus disease 2019, thrombosis, bleeding, death

## Abstract

Background: COVID-19 patients are at high thrombotic risk. The safety and efficacy of different anticoagulation regimens in COVID-19 patients remain unclear. Methods: We searched for randomised controlled trials (RCTs) comparing intermediate- or therapeutic-dose anticoagulation to standard thromboprophylaxis in hospitalised patients with COVID-19 irrespective of disease severity. To assess efficacy and safety, we meta-analysed data for all-cause mortality, clinical status, thrombotic event or death, and major bleedings. Results: Eight RCTs, including 5580 patients, were identified, with two comparing intermediate- and six therapeutic-dose anticoagulation to standard thromboprophylaxis. Intermediate-dose anticoagulation may have little or no effect on any thrombotic event or death (RR 1.03, 95% CI 0.86–1.24), but may increase major bleedings (RR 1.48, 95% CI 0.53–4.15) in moderate to severe COVID-19 patients. Therapeutic-dose anticoagulation may decrease any thrombotic event or death in patients with moderate COVID-19 (RR 0.64, 95% CI 0.38–1.07), but may have little or no effect in patients with severe disease (RR 0.98, 95% CI 0.86–1.12). The risk of major bleedings may increase independent of disease severity (RR 1.78, 95% CI 1.15–2.74). Conclusions: Certainty of evidence is still low. Moderately affected COVID-19 patients may benefit from therapeutic-dose anticoagulation, but the risk for bleeding is increased.

## 1. Introduction

In its severe form, COVID-19, the clinical manifestation associated with SARS-CoV-2 infection, is characterized by respiratory failure and high rates of thromboembolic complications [1]. Procoagulant markers, such as elevated D-Dimers are now widely accepted as prognostic factors for severe disease progression [2,3]. Therefore, a large number of clinical trials aiming to improve outcomes for COVID-19 patients with antithrombotic therapy have begun. For selected hospitalised medical and surgical non-COVID patients, prophylactic low-dose anticoagulation, typically with low molecular weight heparins or unfractionated heparin, has proven beneficial effects in several randomised prospective studies and is recommended by various national guidelines [4,5]. These recommendations have been widely adopted to COVID-19 patients. A recent systematic review on viscoelastic techniques, namely thromboelastography and thromboelastometry, showed that severe COVID-19 is further associated with fibrinolysis shutdown and hyperfibrinogenaemia, despite the use of appropriate thromboprophylaxis [6]. Given the pro-coagulant status and increased thrombotic risk of COVID-19, the question remains whether intensified prophylactic anticoagulation with intermediate or therapeutic doses can decrease the risk of disease progression, clinical worsening or death without increasing the risk for adverse events, such as major bleedings [7]. We therefore performed a systematic review with meta-analysis of available randomised-controlled trials (RCTs) to determine the safety and efficacy of intensified anticoagulation with intermediate or therapeutic doses compared to standard-dose thromboprophylaxis in hospitalised patients with COVID-19.

## 2. Materials and Methods

The original review protocol for this review was registered with PROSPERO on 21 January 2021 (CRD42021229228). Pre-specified outcomes in the protocol have been changed to address competing risk issues. Clinical worsening and thrombotic outcomes now include death as an event. Reporting of this systematic review followed PRISMA [8].

### 2.1. Criteria for Considering Studies for This Review

We considered RCTs investigating thromboprophylaxis in hospitalised patients with a confirmed diagnosis of COVID-19 (RT-PCR or antigen testing) irrespective of age, gender, ethnicity, and disease severity for inclusion. Studies in outpatients were excluded. We further excluded non-randomised studies. 

There was no restriction on the type of anticoagulation used. All heparinoids, vitamin-K-antagonists, and direct anticoagulants (factor Xa inhibitors and direct thrombin inhibitors) were eligible, independent from dosage and regimen. Dosing schemes of anticoagulants were categorised into low, intermediate, and therapeutic doses according to trials’ definition and general drug recommendations [9], which are summarized in Table S1. We included all trials comparing any intermediate- or therapeutic-dose anticoagulation to a control intervention, e.g., standard of care thromboprophylaxis, placebo, no intervention or different prophylactic anticoagulant (same dose category). Standard of care thromboprophylaxis was defined as a low-dose anticoagulation with low molecular weight heparin (LMWH) or unfractionated heparin (UFH) in hospitalised patients with COVID-19 (in the absence of any contraindications). During this pandemic national practice guidelines changed to recommend that COVID-19 patients with advanced respiratory support are considered for intermediate-dose instead of low-dose anticoagulation [10]. Therefore, we extended our definition of standard thromboprophylaxis, including low- and intermediate-dose anticoagulation regimens, and created the following comparisons for meta-analyses:

- Therapeutic-dose versus standard thromboprophylaxis (low- or intermediate-dose anticoagulation);
- Intermediate-dose versus low-dose anticoagulation.

Patient-relevant core outcomes were continuously evolving during this pandemic and were evaluated in accordance with the Core Outcome Measures in Effectiveness Trials (COMET) Initiative for COVID-19 patients [11,12], and additional outcomes that have been prioritised by consumer representatives and the German guideline panel for treatment of hospitalised COVID-19 patients. Outcomes were in line with a series of Cochrane Reviews investigating treatments and therapies for COVID-19 [13,14].

Efficacy of treatment:

- All-cause mortality at day 28, day 60, time-to-event, and at hospital discharge;
- Clinical status at day 28, day 60, and up to the longest follow-up, including:
  ∘ Worsening of clinical status: participants with clinical deterioration (e.g., new need for invasive mechanical ventilation) or death;
  ∘ Improvement of clinical status: participants discharged alive. Participants should be discharged without clinical deterioration or death.
- Any thrombotic event or death within 28 days;
- Any thrombotic event within 28 days;
- Quality of life, including fatigue and neurological status, assessed with standardised scales (e.g., WHOQOL-100) at up to 7 days, up to 28 days, and longest follow-up available;

Safety of treatment:

- Serious adverse events during the study period, defined as number of participants with any event;
- Adverse events (any grade) during the study period, defined as number of participants with any event;
- Major bleeding (ISTH criteria [15]) during the study period.

### 2.2. Search Methods for Identification of Studies

We searched the Cochrane COVID-19 Study Register (comprising MEDLINE, Embase, ClinicalTrials.gov, WHO International Clinical Trials Registry Platform, medRxiv, and the Cochrane Central Register of Controlled Trials), Web of Science (Emerging Citation Index and Science Citation Index), WHO COVID-19 Global literature on coronavirus disease, and Research Square to identify completed and ongoing studies to 24 September 2021. Details on the search strategies are available in the supplementary materials.

### 2.3. Data Collection and Analyses

Three review authors (SR, MP, SW) independently assessed eligible studies in the process of study selection. We conducted data extraction according to the guidelines proposed by Cochrane [16]. Two out of three review authors (SR, MP, SW) extracted data independently and in duplicate, using a customised data extraction form developed in Microsoft Excel. Discrepancies were resolved by discussion between the review authors.

We used the risk of bias 2 (RoB 2) tool to analyse the risk of bias of study results contributing information to our outcomes [17]. Also, the effect of the assignment to the intervention was of interest (the intention-to-treat (ITT) effect). Thus, we performed all assessments with RoB 2 on this effect. Three review authors (SR, MP, SW) independently assessed the risk of bias for each outcome. We assessed the following types of bias as outlined in Chapter 8 of the Cochrane Handbook for Systematic Reviews of Interventions [18]: Bias arising from the randomisation process, deviations from the intended interventions, missing outcome data, measurement of the outcome, selection of the reported result. Subsequently, we derived an overall risk of bias rating for each pre-specified outcome in each study (low risk of bias, some concerns, high risk of bias).

For dichotomous outcomes, we recorded the number of events and total number of participants in both treatment and control groups. We reported the risk ratio (RR) with a 95% confidence interval (CI).

We performed meta-analyses according to the recommendations of the Cochrane Handbook for Systematic Reviews of Interventions [19]. If clinical and methodological characteristics of individual studies were sufficiently homogeneous, we pooled the data in meta-analysis. We collected information on outcomes from all time points reported in the publications. If only a few studies contributed data to an outcome, we pooled different time points, provided the studies have produced valid data and pooling was clinically reasonable. Random effects meta-analyses were performed with RevMan Web 3.11.1 [20]. Fixed-effect meta-analyses were performed as sensitivity analysis. For hospitalised individuals with moderate or severe COVID-19, we performed subgroup analyses independent of heterogeneity and number of studies according to the severity of the disease at baseline. We considered it essential to test the effect of intervention for its impact in different stages of the disease: moderate (WHO 4 to 5) versus severe disease (WHO 6 to 9) as defined by the WHO Clinical Progression Scale [12]. Studies providing data only for a mixed population including moderate and severe participants were included in the subgroup ‘moderate to severe disease’ (WHO 4 to 9). Statistical heterogeneity was defined as *p* < 0.1 for the Chi^2^ test of heterogeneity or I^2^ ≥ 50%. We had planned to explore heterogeneity by subgroup analysis to calculate RR or MD in conjunction with the corresponding CI for each subgroup, if sufficient studies had been available (at least 10 studies per outcome). For the current review, there were not enough studies available. We planned to investigate risk of reporting bias (publication bias) in pairwise meta-analyses using contour-enhanced funnel plots, when there were 10 or more relevant studies pooled in a meta-analysis. In the current review, there are no meta-analyses including 10 or more studies.

The quality of evidence was assessed using the GRADE (Grading of Recommendations, Assessment, Development and Evaluations) approach [21]. GRADE has four levels of certainty in the evidence: very low, low, moderate, and high. We downgraded our certainty of evidence one or two levels for risk of bias, imprecision, inconsistency, indirectness, and probability of publication bias.

## 3. Results

**Figure 1:**
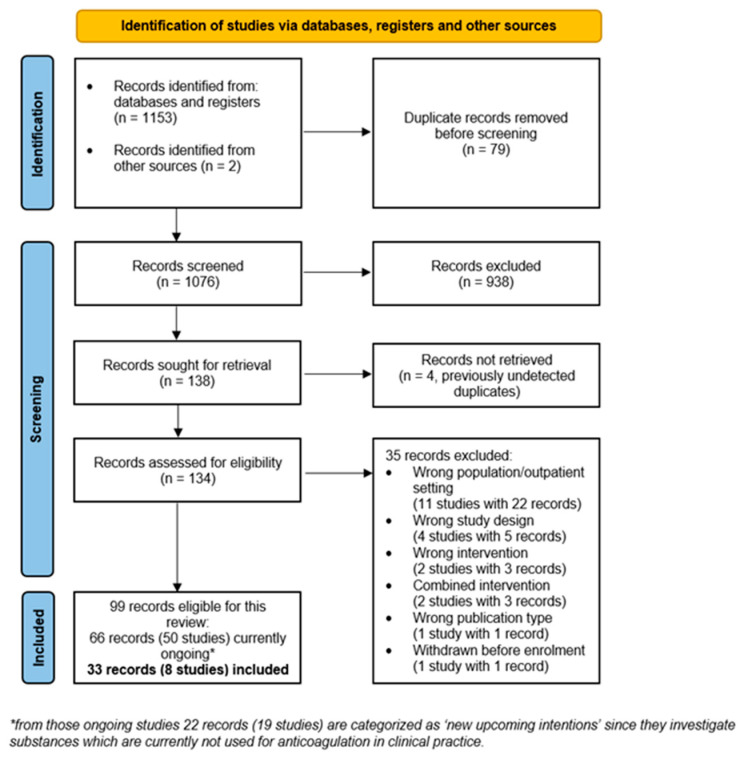
PRISMA flow chart [8].

The search strategy identified a total of 1153 records, two records were identified from other sources. After removal of duplicates, 1076 titles were screened by two authors and assessed for relevance. Full-text screening of 134 records revealed 99 records for inclusion of which 66 (50 studies) are currently ongoing and 35 records (21 studies) were excluded. Eight studies with 33 records were included in this review. Reasons for exclusion of records are summarised in Figure 1.

### 3.1. Study Characteristics

We identified eight RCTs with 5580 randomised COVID-19 participants investigating thromboprophylaxis in hospital settings, all of which were open-label (Table 1) [22,23,24,25,26,27,28,29,30]. The patients’ populations in ATTACC, ACTIV-4a, REMAP-CAP non-critically ill platform trial with 2244 participants and the RAPID trial with 465 participants consisted of over 94% moderate COVID-19 patients (WHO 4–5) [24,29]. According to the WHO scale, only 5% and 6% of participants in both trials were severely affected. We therefore classified both trials´ populations as moderate COVID-19 (WHO 4–5). Two studies, the HESA-COVID trial with 20 participants and the ATTACC, ACTIV-4a, REMAP-CAP critically ill platform trial with 1207 participants focused on patients with severe COVID-19 (WHO 6–9) [23,25]. Four studies included participants with a mixed population of moderate to severe COVID-19 at baseline (WHO 4–9) [22,26,27,28,29,30]. In the ACTION trial with 614 subjects, 85% of the participants had moderate COVID–19 and 15% presented its severe form [26,29]. The INSPIRATION trial with 600 participants included ICU patients, however, according to the WHO scale, 45% of participants were only moderately affected requiring oxygen support by nasal cannula, face, or reservoir mask (WHO 5). We therefore categorised the trial as mixed population (WHO 4–9) [22,28]. The Perepu-2021 trial with 173 participants included hospitalised participants but did not report any details on respiratory support or organ support at baseline [27]. The HEP-COVID trial with 257 participants presented data at least for some outcomes for different strata according to WHO 5 and WHO 6–7 [30].

Three studies had markers for hypercoagulability and coagulopathy as selection criteria. The ACTION trial only included participants with D-Dimer elevation [26], Perepu-2021 trial included participants with a modified ISTH Overt DIC score ≥ 3 [27], and HEP-COVID trial included participants with D-Dimer elevation or ISTH SIC score ≥ 4 [30]. The International Society on Thrombosis and Haemostasis (ISTH) overt disseminated intravascular coagulation (DIC) score focuses on fibrin-specific markers, such as D-Dimer levels, platelet count, fibrinogen levels and prothrombin time [31]. The SIC (sepsis induced coagulopathy) score, developed to identify the predecessor of DIC, considers platelet count, prothrombin time, and SOFA (sequential organ failure assessment) score [31].

Two studies, INSPIRATION and Perepu-2021, with 773 randomised patients investigated intermediate-dose anticoagulation (enoxaparin 1 mg/kg OD) compared to standard low-dose thromboprophylaxis [22,27,28]. All other studies with 4807 patients examined therapeutic-dose anticoagulation vs standard (low- or intermediate-dose) thromboprophylaxis [23,24,25,26,29,30]. Due to updated national treatment guidelines in the UK, the platform trials increased standard thromboprophylaxis during the study period from low- to intermediate-dose anticoagulation in the comparator arm [23,24]. Apart from one study that investigated rivaroxaban as intervention [26], all other studies focused on LMWH (mainly enoxaparin) as anticoagulant [22,23,24,25,27,28,29,30]. Therapeutic anticoagulation was achieved with either enoxaparin 1 mg/kg OD/BID, rivaroxaban 20 mg OD, UFH according to target anti Xa concentration or aPTT, or LMWH according to local protocols for the treatment of acute VTE. Standard thromboprophylaxis was commonly defined as UFH 5000IE two to three times daily or enoxaparin 40 mg OD. Dosing was adjusted for weight and/or creatinine clearance.

Outcomes of interest for this review were reported in most of the studies and the period for outcome assessment was 28–30 days. INSPIRATION reported mortality in the long term at 90 days in a secondary publication [22]. No study reported data for the outcomes’ quality of life, any adverse event, and any serious adverse event.

### 3.2. Risk of Bias

**Figure 2:**
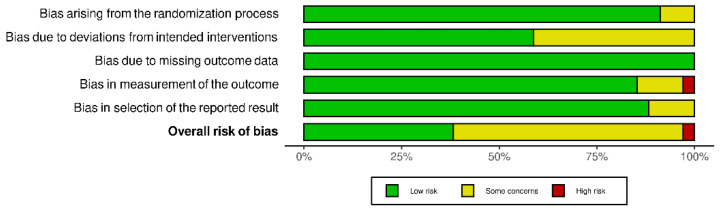
RoB 2 judgements for all domains [32].

In total, the seven studies contributed 34 study results to 14 outcomes, five for the comparison ‘intermediate-dose vs standard-dose’ and nine for the comparison ‘therapeutic-dose vs low-/intermediate-dose’. About one third of the 34 study results (38.2%) were assessed as overall low risk of bias. Of the remaining study results, 58.8% were assessed as some concerns for the overall risk of bias and one (2.9%) as overall high risk of bias (Figure 2). The RoB 2 judgements for all study results per outcome and for all domains are available in Supplementary Material Figures S1 and S2.

### 3.3. Intermediate-Dose Anticoagulation

INSPIRATION [21,27] and Perepu 2021 [27] were included in the comparison of intermediate-dose anticoagulation versus standard thromboprophylaxis with low-dose anticoagulation (Table 2). Intermediate-dose anticoagulation compared to standard thromboprophylaxis in patients with moderate to severe COVID-19 may have little or no effect on all-cause mortality at 30 days (RR 0.98, 95% CI 0.74–1.32, 763 participants, two studies, low-certainty evidence) and 90 days (RR 1.07, 95% CI 0.89–1.28, 590 participants, one study, low-certainty evidence). Intermediate-dose anticoagulation compared to standard thromboprophylaxis may have little or no effect within 30 days on any thrombotic events or death (RR 1.03, 95% CI 0.86–1.24, 590 participants, one study, low-certainty evidence) and on any thrombotic events (RR 0.99, 95% CI 0.51–1.96, 763 participants, two studies, low-certainty evidence). Intermediate-dose anticoagulation may increase major bleedings compared to standard thromboprophylaxis (RR 1.48, 95% CI 0.53–4.15, 763 participants, 2 studies, low-certainty evidence). Certainty of evidence was downgraded for all outcomes due to serious risk of bias and serious imprecision (Table 2).

### 3.4. Therapeutic-Dose Anticoagulation

Six studies [23,24,25,26,29,30] were included in the comparison of therapeutic-dose anticoagulation versus standard thromboprophylaxis with low- or intermediate-dose anticoagulation (Table 3). Therapeutic-dose anticoagulation compared to standard thromboprophylaxis may decrease all-cause mortality at 28 days for moderate COVID-19 patients (RR 0.23, 95% CI 0.08–0.67, 465 participants, one study, low-certainty evidence). We are uncertain on the effect in severe COVID-19 patients (RR 0.33, 95% CI 0.04–2.69, 20 participants, one study, very low-certainty evidence). In studies with mixed COVID-19 population, therapeutic-dose anticoagulation may have little or no effect on all-cause mortality at 28 days (RR 1.07, 95% CI 0.56–2.03, 867 participants, two studies, low-certainty evidence). Contrary to effects for all-cause mortality at 28 days, therapeutic-dose anticoagulation may have little or no effect on in-hospital mortality (RR 0.97, 95% CI 0.79–1.19, 3344 participants, three studies, low-certainty evidence) in all hospitalised COVID-19 patients. Subgroup analysis showed similar results irrespective of disease severity.

Therapeutic-dose anticoagulation compared to standard thromboprophylaxis may have little or no effect on worsening of clinical status within 28 days assessed as progression to intubation or death in one study with patients affected by moderate COVID-19 (RR 0.90, 95% CI 0.72–1.14, 2231 participants, one study, low-certainty evidence). It may decrease the progression to any mechanical ventilation or death assessed in another study with moderate COVID-19 participants (WHO 4–9) (RR 0.63, 95% CI 0.39–1.02, 465 participants, one study, low-certainty evidence).

Therapeutic-dose anticoagulation compared to standard thromboprophylaxis has no effect on the improvement of clinical status assessed as participants discharged alive without clinical worsening at 28 days assessed in one study with moderate to severe COVID-19 patients (0.96, 95% CI 0.90–1.02, 614 participants, one study, high-certainty evidence). Another study with moderate COVID-19 patients only, found that therapeutic anticoagulation, may slightly increase the improvement of clinical status defined as discharge without receiving organ support within 28 days (RR 1.05, 95% CI 1.00–1.10, 2219 participants, one study, low-certainty evidence).

**Figure 3:**
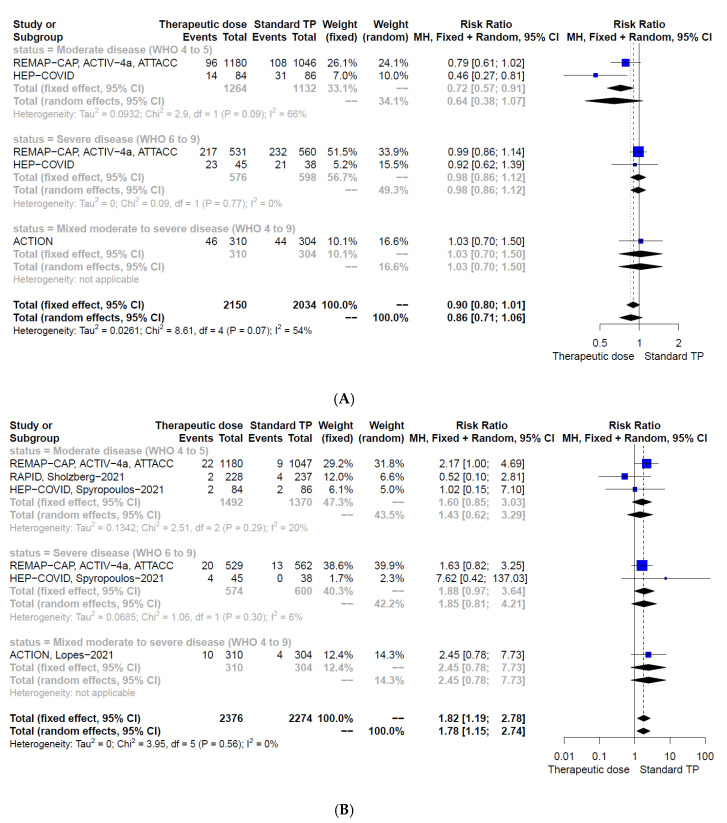
Forest plots according to pre-specified subgroups (moderately and severely diseased population) of therapeutic-dose anticoagulation vs. standard-dose thromboprophylaxis for the outcomes (**A**) any thrombotic event or death at 28 to 30 days and (**B**) major bleedings; CI, confidence interval; M–H, Mantel–Haenszel; TP, thromboprophylaxis.

Therapeutic-dose anticoagulation may have little or no effect on any thrombotic event or death within 28 days in pooled meta-analysis including all hospitalised COVID-19 participants irrespective of disease severity (RR 0.86, 95% CI 0.71–1.06, 4184 participants, four studies, low-certainty evidence, Figure 3A). Subgroup analysis showed that in patients with moderate COVID-19 therapeutic-dose anticoagulation compared to standard thromboprophylaxis may decrease any thrombotic event or death (RR 0.64, 95% CI 0.38–1.07, 2396 participants, two studies, low-certainty evidence, Figure 3A). This effect was statistically significant with fixed effect meta-analysis (RR 0.72, 95% CI 0.57–0.91, Figure 3A). In participants with severe COVID-19, therapeutic-dose anticoagulation may have little or no effect on any thrombotic event or death (RR 0.98, 95% CI 0.86–1.12, 1174 participants, two studies, low-certainty evidence, Figure 3A). Therapeutic-dose anticoagulation compared to standard thromboprophylaxis may decrease the incidence of any thrombotic event within 28 days (RR 0.58, 95% CI 0.45–0.74, 4669 participants, six studies, moderate-certainty evidence). Subgroup analysis showed similar results irrespective of disease severity.

Therapeutic-dose anticoagulation compared to standard thromboprophylaxis may increase major bleedings within 30 days irrespective of disease severity (RR 1.78, 95% CI 1.15–2.74, 4650 participants, five studies, low-certainty evidence, Figure 3B).

Certainty of evidence was downgraded for all outcomes except for clinical status assessed as ‘participants discharged alive without clinical worsening’ (Table 3). For all other outcomes, certainty of evidence was downgraded due to serious risk of bias, indirectness, imprecision, or heterogeneity (Table 3). Indirectness was defined in this context as use of mixed low to intermediate-dose anticoagulation in the standard thromboprophylaxis comparator group in two studies [23,24].

## 4. Discussion

In the past, several retrospective observational studies have provided evidence on the potential benefit of therapeutic-dose anticoagulation in COVID-19 patients [33,34]. A meta-analysis of observational studies on 25,719 hospitalised COVID-19 patients showed that anticoagulant use was associated with more than 50% reduction of in-hospital mortality, particularly in ICU patients [35]. Effects of this strength must be examined and confirmed in high-quality RCTs and systematic review with meta-analysis to inform guideline committees and clinicians.

Our meta-analysis, including 773 hospitalised COVID-19 participants from two RCTs [22,27,28], did not show any benefit of intermediate-dose anticoagulation compared to standard thromboprophylaxis regarding the outcomes of all-cause mortality at 30 and 90 days, and the risk of any thrombotic event or death. Intermediate-dose anticoagulation may increase risk for major bleeding events, which was not yet statistically significant.

For therapeutic-dose anticoagulation, evidence from six studies with 4807 hospitalised patients with moderate to severe COVID-19 was available [23,24,25,26,29,30]. Data on all-cause mortality measured at 28 days and in-hospital showed little or no effect on the pooled population of patients with moderate and severe COVID-19. However, for the subgroup of patients with moderate COVID-19, therapeutic anticoagulation showed benefit for all-cause mortality at 28 days based on the RAPID trial with 465 participants, whereas, little or no effect was shown for in-hospital mortality based on the large ATTACC, ACTIV-4a, REMAP-CAP platform trial with 2226 participants [24,29]. Both studies, the RAPID and the ATTACC, ACTIV-4a, and REMAP-CAP platform trial also showed conflicting results for their clinical worsening outcomes [24,29]. Similar to mortality, RAPID indicated a benefit in terms of a decreased number of patients with progression to any mechanical ventilation or death due to therapeutic anticoagulation, whereas the platform trial showed little or no effect on progression to intubation or death [24,29]. Certainty of evidence for these conflicting results was low as both trials bear limitations. RAPID was a smaller trial with few events and in the ATTACC, ACTIV-4a, and REMAP-CAP platform trial only 79.6% of non-critically ill participants in the intervention group received the assigned therapeutic-dose anticoagulation, while the rest received anticoagulation at lower dosages. It cannot be excluded that this has influenced the results leading to lower differences between control and intervention groups, especially as a significant proportion of participants in the control group ended up not receiving their assigned usual-care low-dose thromboprophylaxis but an intensified intermediate-dose (26.5% of non-critically ill) due to changes in the national treatment guideline [10,24,29]. Further studies are needed to solve these conflicts.

Our results suggest that therapeutic anticoagulation might not improve the clinical status of COVID-19 patients measured three to four weeks after anticoagulation has been started. In the ACTION trial with 614 participants of moderate and severe COVID-19, improvement of the clinical status was measured as discharged alive without clinical deterioration or death [26]. With high-certainty evidence, therapeutic anticoagulation has no effect on improvement of clinical status and more than 80% of participants in both groups had improved. In the ATTACC, ACTIV-4a, and REMAP-CAP platform trial with moderate COVID-19 patients only, improvement of clinical status was minimal with an absolute difference of 3.8% between the groups and not significant in frequentist analysis without adjustments [24]. Study authors, however, reported significant results using an adjusted Bayesian cumulative logistic model [24]. Independent of the model used, clinical relevance of the absolute effect should be discussed among clinicians.

As to be expected, therapeutic-dose anticoagulation decreased the rate of thrombotic events for all patients independent of disease severity. This outcome was reported in all studies. However, the composite outcome of any thrombotic event or death treating the individual outcomes as competing risks, revealed a reduced risk due to therapeutic anticoagulation only for patients with moderate COVID-19. Additionally, this effect was only statistically significant when using the fixed-effects model. In contrast, for patients with severe COVID-19, there was no effect due to therapeutic anticoagulation shown in the meta-analysis. The effect could be explained by recent findings on COVID-19 associated coagulopathy, suggesting a pro-coagulant state only at the beginning of the infection, which transforms into disseminated intravascular coagulation with increased haemorrhagic risk as the disease progresses [36].

Irrespective of disease severity, therapeutic-dose anticoagulation showed a non-significantly higher rate of major bleeding events in the subgroups, and the pooled effect with a sufficient number of events and patients reached statistical significance. Therefore, the risk for bleeding should be taken into account in decision making and anticoagulated COVID-19 patients should be carefully monitored for bleeding events.

Despite this meta-analysis including evidence from 5580 participants, the overall certainty of evidence for intensified thromboprophylaxis in hospitalised patients with COVID-19 remains low. Limitations of the evidence base are the conflicting results and lack of evidence that would warrant high certainty, due to the wide heterogeneity of study settings, populations, and therapeutic approaches.

The fact that disease severity was not defined in a standardised way throughout different studies hampers straight forward subgroup analysis. Whereas some study results indicate a difference in efficacy of anticoagulation between ICU and general ward, other studies did not make this differentiation in their trial cohort. As ICU settings worldwide vary greatly and are difficult to compare, we adopted the WHO clinical progression scale as a measure of disease severity and classified patient status in trials accordingly [12]. This may have led to inaccuracies as Perepu did not report on the respiratory status and RAPID and the ATTAC, ACTIV-4a, and REMAP-CAP non-critically ill both included a small percentage of severe COVID-19 patients in an otherwise moderately affected cohort [24,27,29].

Prophylactic and therapeutic dosages were not defined in a standardised way in the studies. Whereas some studies adjusted for weight or BMI, others did not, or did so in a different fashion, leading to various dosing regimens. Furthermore, as a result of prompt reaction to new emerging evidence during the pandemic, evolving national treatment guidelines in the UK led to a significant proportion of participants in the large platform trials ATTAC, ACTIV-4a, and REMAP-CAP not receiving their assigned usual-care low-dose thromboprophylaxis but an intensified intermediate-dose anticoagulation (26.5% of non-critically ill, 51.7% of critically ill) [23,24]. Together with the deviation from intended intervention it cannot be excluded, that this has influenced the results, leading to smaller differences between control and intervention groups. Reasons for this deviation were not given by the authors and this aspect was recognised in our risk of bias assessment. The trials also used different types of anticoagulants. The ACTION trial used rivaroxaban 20 mg, which is licensed neither as prophylactic- nor therapeutic-dose for treatment initiation. The used dose of 20 mg is recommended only for long-term treatment of deep venous thrombosis (DVT), treatment of pulmonary embolism (PE) and prophylaxis of recurrent DVT and PE [26,37]. Taking into consideration that heparin has not only an anticoagulant but also an antiviral and anti-inflammatory effect, it cannot be excluded that the results became less pronounced even though clinical relevance is not well established [38].

As the capacity for proper assessment of thromboembolic events during the pandemic was limited in many places, several studies reported confirmed or suspected cases of thrombosis. Whether this led to over- or underreporting of events is unclear. We accounted for this inaccuracy in our risk of bias assessment.

In a recent preprint article, the retrospective analysis of hospital-based records of 168 COVID-19 patients showed that elevated D-Dimers, SIC, and DIC score may be used as predictors of COVID-19 severity [39]. Whether these parameters can be used to guide the anticoagulation strategy in COVID-19 is currently under debate. Three of our included studies, ACTION, Perepu and HEP-COVID, limited their patient population to participants with known activation of the haemostatic and thrombolytic system, such as elevated D-Dimers, SIC and DIC scores. Results in these studies were not different to the other studies. A narrative review recently proposed an algorithm for the anticoagulation strategy based on disease severity and suggested intermediate- or therapeutic-dose anticoagulation for severe COVID-19 cases dependent on the ISTH-DIC score [40]. So far, our meta-analysis also suggests an algorithm for the anticoagulation strategy based on disease severity. However, the population of highest benefit may not be patients with severe COVID-19, but patients with moderate COVID-19.

In conclusion, certainty of evidence on whether intermediate- or therapeutic-dose anticoagulation compared to standard thromboprophylaxis is beneficial or not is still low for hospitalised COVID-19 patients. Results of this meta-analysis and primary studies indicate that moderately affected COVID-19 patients may benefit from therapeutic-dose anticoagulation, but not patients with severe COVID-19. The risk for bleedings is increased independent of disease severity.

## Figures and Tables

**Table 1:** Study characteristics of the eight included trials.

Study Reference	Study Design	Setting and Patient Status	Randomised Patients	Intervention	Comparator	Selected Outcomes
INSPIRATION, [22,28]	RCT, open-label, multi-centre	ICU WHO 5–9, with 45% WHO 5	600	Intermediate-dose anticoagulation ^A^ with enoxaparin 1 mg/kg OD sc for 30 days; weight and CrCI adjusted	Standard thromboprophylaxis with enoxaparin 40 mg OD; weight and CrCI adjusted	30-day mortality, 90-day mortality, any venous thrombotic events, any venous thrombotic events or death, major bleeding
Perepu-2021 [27]	RCT, multi-centre, open-label	Hospitalised + mod. ISTH Overt DIC score ≥3 + ICU, WHO 5–9, no details on respiratory status reported	173	Intermediate-dose anticoagulation ^A^ with enoxaparin 1 mg/kg sc OD until hospital discharge; weight and CrCI adjusted	Standard thromboprophylaxis with enoxaparin 40 mg sc OD until hospital discharge or extended beyond, weight and CrCI adjusted	30-day mortality, any venous thrombotic events, major bleeding
HESACOVID, [25]	RCT, open-label, single centre	ICU WHO ≥ 7	20	Therapeutic-dose anticoagulation ^A^ with enoxaparin 1 mg/kg sc BID for at least 96 h and up to 14 days	Standard thromboprophylaxis with enoxaparin 40 mg OD; weight and CrCI adjusted	28-day mortality, in-hospital mortality, any thrombotic event
ACTION [26]	RCT, multi-centre, open-label	Hospitalised/ ICU + ↑ D-Dimer, WHO 4–9, with 85% WHO 4–5	614	Therapeutic-dose anticoagulation ^A^ with rivaroxaban 20 mg po OD (280 patients, 90%) for 30 days	Standard thromboprophylaxis with enoxaparin 40 mg sc OD, continued until or extended beyond hospital discharge; weight and CrCI adjusted	30-day mortality, survival until hospital discharge (30 days), any thrombotic event, any thrombotic event or death, major bleeding
RAPID 2021 [29]	RCT, multi-centre, open-label	Hospitalised + ↑ D-Dimer, WHO 4–5, with 6% WHO 6	465	Therapeutic-dose anticoagulation ^A^ Enoxaparin 1 mg/kg sc BID; weight and CrCI adjusted	Standard thromboprophylaxis with enoxaparin 40 mg OD, weight and CrCl adjusted	All-cause mortality, venous thrombotic events, major bleeding
ATTACC, ACTIV-4a, REMAP-CAP Non-critically ill [24]	RCT, open-label, Bayesian, adaptive, multiplatform	Hospitalised WHO 4–5, with 5% WHO 6–7	2244	Therapeutic-dose anticoagulation ^A^ (79.6%) with enoxaparin 1 mg/kg sc minus 10% BID, weight and CrCl adjusted	Standard low- or intermediate-dose thromboprophylaxis with 78.7 %: enoxaparin, 9.6%: dalteparin; Low-dose: 71.7%, intermediate-dose: 26.5% subtherapeutic-dose: 0.8% therapeutic-dose: 0.9%	In-hospital mortality, clinical worsening: intubation or death, clinical improvement: discharged without receiving organ support, any thrombotic event, any thrombotic event or death, major bleeding
ATTAC, ACTIV-4a, REMAP-CAP Critically ill [23]	RCT, open-label, Bayesian, adaptive, multiplatform	ICU WHO 6–9, 1.5% WHO 4–5	1207	Therapeutic-dose anticoagulation ^A^ (77.6%) with enoxaparin 1 mg/kg minus 10% BID, weight and CrCl adjusted	Standard low- or intermediate-dose thromboprophylaxis with 52.1%: enoxaparin, 32.8%: dalteparin; Low-dose: 40.4%, Intermediate-dose: 51.7% Subtherapeutic-dose: 1.8% Therapeutic-dose: 6.1%	In-hospital mortality, any thrombotic event, any thrombotic event or death, major bleeding
HEP-COVID 2021 [30]	RCT, multi-center, open-label	Hospitalised + ↑ D-Dimer or ISTH SIC score ≥ 4, WHO 5–7, with 77% WHO 5, both strata reported for some outcomes	257	Therapeutic-dose anticoagulation ^A^ with enoxaparin 1 mg/kg sc BID, or 40 mg sc OD/BID weight and CrCI adjusted, until hospital discharge	Standard thromboprophylaxis with enoxaparin 40 mg sc OD/BID weight and CrCI adjusted, until hospital discharge	All-cause mortality, any thromboembolic event, any thromboembolic event or death, major bleeding

RCT, randomised controlled trial; ICU, intensive care unit; sc, sub-cutaneous; OD, once daily; BID, twice daily; UFH, unfractionated heparin; CrCl, creatinine clearance; +, plus; ↑, elevated. ^A^ Defined according to trial protocol.

**Table 2:** Meta-analyses for intermediate-dose anticoagulation versus standard thromboprophylaxis, including certainty of evidence.

Outcome	Study Population *	Risk Ratio (M–H, Random, 95% CI)	Risk Ratio (M–H, Fixed, 95% CI)	Heterogeneity	Certainty of Evidence
All-cause mortality at 30 days	Pooled effect, mixed population (WHO 4–9), 763 participants, 2 studies [22,27,28]	0.98 (0.74, 1.32)	1.01 (0.84, 1.21)	Tau^2^ = 0.02; Chi^2^ = 1.28, df = 1 (*p* = 0.26); I^2^ = 22%	Low-certainty evidence due to serious risk of bias and imprecision
All-cause mortality at 90 days	Mixed population (WHO 4–9), 590 participants, 1 study [22,28]	1.07 (0.89, 1.28)	1.07 (0.89, 1.28)	NA	Low-certainty evidence due to serious risk of bias and imprecision
Any thrombotic event or death up to 30 days	Mixed population (WHO 4–9), 590 participants, 1 study [22,28]	1.03 (0.86, 1.24)	1.03 (0.86, 1.24)	NA	Low-certainty evidence due to serious risk of bias and imprecision
Any venous thrombotic event up to 30 days	Pooled effect, mixed population (WHO 4–9), 763 participants, 2 studies [22,27,28]	0.99 (0.51, 1.96)	0.99 (0.50, 1.95)	Chi^2^ = 0.13, df = 1 (*p* = 0.72); I^2^ = 0%	Low-certainty evidence due to serious risk of bias and imprecision
Major bleeding up to 28 days	Pooled effect, mixed population (WHO 4–9), 763 participants, 2 studies [22,27,28]	1.48 (0.53, 4.15)	1.49 (0.53, 4.14)	Tau^2^ = 0.00; Chi^2^ = 0.23, df = 1 (*p* = 0.63); I^2^ = 0%	Low-certainty evidence due to serious risk of bias and imprecision

M–H, Mantel–Haenszel; CI, confidence interval. * Patient status according to WHO clinical progression scale.

**Table 3:** Meta-analyses for therapeutic-dose anticoagulation according to pre-specified subgroups (moderate population and severe population) including certainty of evidence.

Outcome	Study Population *	Risk Ratio (M–H, Random, 95% CI)	Risk Ratio (M–H, Fixed, 95% CI)	Heterogeneity	Certainty of Evidence
All-cause mortality (28 days)	Moderately diseased population (WHO 4–5), 465 participants, 1 study [29]	0.23 (0.08, 0.67)	0.23 (0.08, 0.67)	NA	Low-certainty evidence due to very serious imprecision
	Severely diseased population (WHO 6–9), 20 participants, 1 study [25]	0.33 (0.04, 2.69)	0.33 (0.04, 2.69)	NA	Very low-certainty evidence due to risk of bias and very serious imprecision
	Mixed population (WHO 4–9), 867 participants, 2 studies [26,30]	1.07 (0.56, 2.03)	1.08 (0.77, 1.51)	Tau^2^ = 0.16; Chi^2^ = 3.54, df = 1 (*p* = 0.06); I^2^ = 72%	Low-certainty evidence due to serious heterogeneity and imprecision
	Pooled effect, mixed population (WHO 4–9), 1352 participants, 4 studies [25,26,29,30]	0.68 (0.32, 1.45)	0.85 (0.62, 1.16)	Tau^2^ = 0.38; Chi^2^ = 11.47, df = 3 (*p* = 0.009); I^2^ = 74%	Low-certainty evidence due to serious heterogeneity and imprecision
All-cause mortality in hospital	Pooled effect, mixed population (WHO 4–9), 3344 participants, 3 studies [23,24,25]	0.97 (0.79, 1.19)	0.99 (0.86, 1.13)	Tau^2^ = 0.01; Chi^2^ = 2.78, df = 2 (*p* = 0.25); I^2^ = 28%	Low-certainty evidence due to serious indirectness and risk of bias
Worsening of clinical status: Progression to intubation or death (28 days)	Moderately diseased population (WHO 4–5), 2231 participants, 1 study [24]	0.90 (0.72, 1.14)	0.90 (0.72, 1.14)	NA	Low-certainty evidence due to serious indirectness and risk of bias
Worsening of clinical status: Progression to any mechanical ventilation or death (28 days)	Moderately diseased population (WHO 4–5), 465 participants, 1 study [29]	0.63 (0.39, 1.02)	0.63 (0.39, 1.02)	NA	Low-certainty evidence due to very serious imprecision
Improvement of clinical status: participants discharged alive without clinical deterioration or death at 28 days	Mixed population (WHO 4–9), 614 participants, 1 study [26]	0.96 (0.90, 1.02)	0.96 (0.90, 1.02)	NA	High-certainty evidence
Improvement of clinical status: survival until hospital discharge without receiving organ support	Moderately diseased population (WHO 4–5), 2219 participants, 1 study [24]	1.05 (1.00, 1.10)	1.05 (1.00, 1.10)	NA	Low-certainty evidence due to serious indirectness and risk of bias
Any thrombotic event or death	Moderately diseased population (WHO 4–5), 2396 participants, 2 studies [24,30]	0.64 (0.38, 1.07)	0.72 (0.57, 0.91)	Chi^2^ = 2.90, df = 1 (*p* = 0.09); I^2^ = 66%	Low-certainty evidence due to serious risk of bias and indirectness/heterogeneity
	Severely diseased population (WHO 6–9), 1174 participants, 2 studies [23,30]	0.98 (0.86, 1.12)	0.98 (0.86, 1.12)	Chi^2^ = 0.09, df = 1 (*p* = 0.77); I^2^ = 0%	Low-certainty evidence due to serious risk of bias and indirectness
	Mixed population (WHO 4–9), 614 participants, 1 study [26]	1.03 (0.70, 1.50)	1.03 (0.70, 1.50)	NA	Low-certainty evidence due to serious risk of bias and imprecision
	Pooled effect, mixed population (WHO 4–9), 4184 participants, 4 studies [23,24,26,30]	0.86 (0.71, 1.06)	0.90 (0.80, 1.01)	Chi^2^ = 8.61, df = 4 (*p* = 0.07); I^2^ = 54%	Low-certainty evidence due to serious risk of bias and indirectness/heterogeneity
Any thrombotic event	Pooled effect, mixed population (WHO 4–9), 4669 participants, 6 studies [23,24,25,26,29,30]	0.58 (0.45, 0.74)	0.57 (0.45, 0.73)	Tau^2^ = 0.00; Chi^2^ = 4.68, df = 5 (*p* = 0.46); I^2^ = 0%	Moderate-certainty evidence due to serious risk of bias
Major bleeding at 28 days	Pooled effect, mixed population (WHO 4–9), 4650 participants, 5 studies [23,24,26,29,30]	1.78 (1.15, 2.74)	1.82 (1.19, 2.78)	Tau^2^ = 0.00; Chi^2^ = 3.95, df = 5 (*p* = 0.56); I^2^ = 0%	Low-certainty evidence due to serious indirectness and risk of bias

M–H, Mantel–Haenszel; CI, confidence interval. * Patient status according to WHO clinical progression scale.

## Data Availability

Extracted data are available on request to the corresponding author.

## Supplementary Materials

The following are available online at https://www.mdpi.com/article/10.3390/jcm11010057/s1; Table S1: Definition of prophylactic- and therapeutic-dose anticoagulation; Table S2: Meta-analyses for therapeutic-dose anticoagulation according to pre-specified subgroups (moderately and severely diseased population) including certainty of evidence; Table S3: PRISMA 2020 checklist; Figure S1: Forest plots of intermediate-dose anticoagulation vs. standard dose thromboprophylaxis; Figure S2: Forest plots according to pre-specified subgroups (moderate and severe population) of therapeutic-dose anticoagulation vs. standard dose thromboprophylaxis; Search strategy.
