# Supplementary material for: Safety and Efficacy of Intermediate- and Therapeutic-Dose Anticoagulation for Hospitalised Patients with COVID-19: A Systematic Review and Meta-Analysis
Source: J Clin Med. 2021 Dec 23;11(1):57. doi: 10.3390/jcm11010057 (PMC8745419; doi:10.3390/jcm11010057)
Supplement: Supplementary file 1 [file jcm-11-00057-s001.zip › jcm-1508070-supplementary.pdf]

Journal of
Clinical Medicine

Supplementary Materials

Table S1 Definition of prophylactic and therapeutic dose anticoagulation.. 2

Table S2 Meta-analyses for therapeutic dose anticoagulation according to pre-specified subgroups (moderate and severe population) including certainty of evidence 2

Table S3 Prisma 2020 checklist.. 4

Figure S1: Forest plots of intermediate dose anticoagulation vs standard dose thromboprophylaxis6

Figure S2: Forest plots according to pre-specified subgroups (moderately and severely diseased population) of therapeutic dose anticoagulation vs standard dose thromboprophylaxis 8

Search strategy.. 12

Table S1. Definition of prophylactic and therapeutic dose anticoagulation.

	Prophylactic dose anticoagulation	Therapeutic dose anticoagulation
**LMWH**	Enoxaparin 40mg sc OD (4)	Enoxaparin 1.0 mg/kg BID (37)
**UFH**	5000IE sc TID (4)	5000IE bolus, followed by 15-20IE/kg/h (37)
**DOAC**	Rivaroxaban licensed neither as prophylactic- nor therapeutic-dose for treatment initiation; 20 mg for long-term treatment of DVT, PE and prophylaxis of recurrent DVT and PE	Rivaroxaban 15mg BID (37)

LMWH, low molecular weight heparin; UFH, unfractionated heparin; DOAC, direct oral anticoagulant; sc, subcutaneously, OD, once daily; BID, twice daily; TID, three times daily, DVT, deep vein thrombosis, PE, pulmonary embolism.

Table S2. Meta-analyses for therapeutic dose anticoagulation according to pre-specified subgroups (moderate and severe population) including certainty of evidence.

Outcome	Study population*	Risk ratio (M-H, random, 95% CI)	Risk ratio (M-H, Fixed, 95% CI)	heterogeneity	Certainty of evidence
All-cause mortality at 28 days	Moderately diseased population (WHO 4–5), 465 participants, 1 study [28]	0.23 [0.08, 0.67]	0.23 [0.08, 0.67]	NA	Low-certainty evidence due to very serious imprecision
	Severely diseased population (WHO 6–9), 20 participants, 1 study [24]	0.33 [0.04, 2.69]	0.33 [0.04, 2.69]	NA	Very low-certainty evidence due to risk of bias and very serious imprecision
	Mixed population (WHO 4–9), 867 participants, 2 studies [25,29]	1.07 [0.56, 2.03]	1.08 [0.77, 1.51]	$Tau^2 = 0.16$; $Chi^2 = 3.54$, df = 1 (P = 0.06); $I^2 = 72\%$	Low-certainty evidence due to serious heterogeneity and imprecision
	Pooled effect, mixed population (WHO 4–9), 1352 participants, 4 studies [24,25,28,29]	0.68 [0.32, 1.45]	0.85 [0.62, 1.16]	$Tau^2 = 0.38$; $Chi^2 = 11.47$, df = 3 (P = 0.009); $I^2 = 74\%$	Low certainty evidence due to serious heterogeneity and imprecision
All-cause mortality in hospital	Moderately diseased population (WHO 4–5), 2226 participants, 1 study [27]	0.89 [0.67, 1.18]	0.89 [0.67, 1.18]	NA	NA
	Severely diseased population (WHO 6–9), 1118 participants 2 studies [25,26]	0.84 [0.37, 1.87]	1.03 [0.89, 1.21]	$Tau^2 = 0.21$; $Chi^2 = 1.84$, df = 1 (P = 0.17); $I^2 = 46\%$	NA
	Pooled effect, mixed population (WHO 4–9), 3344 participants, 3 studies [22–24]	0.97 [0.79, 1.19]	0.99 [0.86, 1.13]	$Tau^2 = 0.01$; $Chi^2 = 2.78$, df = 2 (P = 0.25); $I^2 = 28\%$	Low certainty evidence due to serious indirectness and risk of bias
Worsening of clinical status: Progression to	Moderately diseased population (WHO 4–	0.90 [0.72, 1.14]	0.90 [0.72, 1.14]	NA	Low certainty evidence due to serious indirectness and risk of bias

intubation at 28 days	5), 2231 participants, 1 study [27]				
Worsening of clinical status: Progression to any mechanical ventilation or death (28 days)	Moderately diseased population (WHO 4–5), 465 participants, 1 study [28]	0.63 [0.39, 1.02]	0.63 [0.39, 1.02]	NA	Low certainty evidence due to serious imprecision
Improvement of clinical status: participants discharged alive without clinical deterioration or death at 28 days	Mixed population (WHO 4–9), 614 participants, 1 study [24]	0.96 [0.90, 1.02]	-0.03 [−0.09, 0.02]	NA	High certainty evidence
Improvement of clinical status: survival until hospital discharge without receiving organ support	Moderately diseased population (WHO 4–5), 2219 participants, 1 study [27]	1.05 [1.00, 1.10]	1.05 [1.00, 1.10]	NA	Low certainty evidence due to serious indirectness and risk of bias
Any thrombotic event or death	Moderately diseased population (WHO 4–5), 2396 participants, 2 studies [23,29]	0.64 [0.38, 1.07]	0.72 [0.57, 0.91]	Chi^2 = 2.90, df = 1 (P = 0.09); I^2 = 66%	Low-certainty evidence due to serious risk of bias and indirectness/heterogeneity
	Severely diseased population (WHO 6–9), 1174 participants, 2 studies [22,29]	0.98 [0.86, 1.12]	0.98 [0.86, 1.12]	Chi^2 = 0.09, df = 1 (P = 0.77); I^2 = 0%	Low-certainty evidence due to serious risk of bias and indirectness
	Mixed population (WHO 4–9), 614 participants, 1 study [25]	1.03 [0.70, 1.50]	1.03 [0.70, 1.50]	NA	Low-certainty evidence due to serious risk of bias and imprecision
	Pooled effect, mixed population (WHO 4–9), 4184 participants, 4 studies [22,23,25,29]	0.86 [0.71, 1.06]	0.90 [0.80, 1.01]	Chi^2 = 8.61, df = 4 (P = 0.07); I^2 = 54%	Low certainty evidence due to serious risk of bias and indirectness/heterogeneity
Any thrombotic event	Moderately diseased population (WHO 4–5), 2691 participants, 2 studies [27,28]	0.47 [0.27, 0.83]	0.47 [0.27, 0.82]	Tau^2 = 0.00; Chi^2 = 0.39, df = 1 (P = 0.53); I^2 = 0%	NA
	Severely diseased population (WHO 6–9), 1109 participants, 2 studies [25,26]	0.66 [0.45, 0.96]	0.66 [0.45, 0.96]	Tau^2 = 0.00; Chi^2 = 0.23, df = 1 (P = 0.63); I^2 = 0%	NA

	Mixed population (WHO 4–9), 869 participants, 2 studies [24,29]	0.54 [0.28, 1.05]	0.55 [0.38, 0.80]	Tau^2 = 0.16; Chi^2 = 3.03, df = 1 (P = 0.08); I^2 = 67%	NA
	Pooled effect, mixed population (WHO 4–9), 4669 participants, 6 studies [22–25,28,29]	0.58 [0.45, 0.74]	0.57 [0.45, 0.73]	Tau^2 = 0.00; Chi^2 = 4.68, df = 5 (P = 0.46); I^2 = 0%	Moderate certainty evidence due to serious risk of bias
Major bleeding at 28 days	Moderately diseased population (WHO 4–5), 2397 participants, 2 studies [27,29]	1.96 [0.96, 4.01]	1.97 [0.97, 4.02]	Tau^2 = 0.00; Chi^2 = 0.50, df = 1 (P = 0.48); I^2 = 0%	NA
	Severely diseased population (WHO 6–9), 1174 participants, 2 studies [26,29]	1.85 [0.81, 4.23]	1.88 [0.97, 3.64]	Tau^2 = 0.07; Chi^2 = 1.06, df = 1 (P = 0.30); I^2 = 6%	NA
	Mixed population (WHO 4–9), 1079 participants, 2 studies [24,28]	1.28 [0.29, 5.75]	1.50 [0.62, 3.66]	Tau^2 = 0.66; Chi^2 = 2.22, df = 1 (P = 0.14); I^2 = 55%	NA
	Pooled effect, mixed population (WHO 4–9), 4650 participants, 5 studies [22,23,25,28,29]	1.78 [1.15, 2.74]	1.82 [1.19, 2.78]	Tau^2 = 0.00; Chi^2 = 3.95, df = 5 (P = 0.56); I^2 = 0%	Low certainty evidence due to serious indirectness and risk of bias

M-H, Mantel-Haenszel; CI, confidence interval. * Patient status according to WHO clinical progression scale

Table S3. PRISMA 2020 checklist.

Section and Topic	Item #	Checklist item	Location where item is reported
TITLE			
Title	1	Identify the report as a systematic review.	Title
ABSTRACT			
Abstract	2	See the PRISMA 2020 for Abstracts checklist.	Abstract
INTRODUCTION			
Rationale	3	Describe the rationale for the review in the context of existing knowledge.	1
Objectives	4	Provide an explicit statement of the objective(s) or question(s) the review addresses.	1
METHODS			
Eligibility criteria	5	Specify the inclusion and exclusion criteria for the review and how studies were grouped for the syntheses.	2.1
Information sources	6	Specify all databases, registers, websites, organisations, reference lists and other sources searched or consulted to identify studies. Specify the date when each source was last searched or consulted.	2.2
Search strategy	7	Present the full search strategies for all databases, registers and websites, including any filters and limits used.	Supplementary materials
Selection process	8	Specify the methods used to decide whether a study met the inclusion criteria of the review, including how many reviewers screened each record and each report retrieved, whether they worked independently, and if applicable, details of automation tools used in the process.	2.3
Data collection process	9	Specify the methods used to collect data from reports, including how many reviewers collected data from each report, whether they worked independently, any processes for obtaining or confirming data from study investigators, and if applicable, details of automation tools used in the process.	2.3
Data items	10a	List and define all outcomes for which data were sought. Specify whether all results that were compatible with each outcome domain in each study were sought (e.g. for all measures, time points, analyses), and if not, the methods used to decide which results to collect.	2.1; 2.3

Section and Topic	Item #	Checklist item	Location where item is reported
	10b	List and define all other variables for which data were sought (e.g. participant and intervention characteristics, funding sources). Describe any assumptions made about any missing or unclear information.	2.3
Study risk of bias assessment	11	Specify the methods used to assess risk of bias in the included studies, including details of the tool(s) used, how many reviewers assessed each study and whether they worked independently, and if applicable, details of automation tools used in the process.	2.3
Effect measures	12	Specify for each outcome the effect measure(s) (e.g. risk ratio, mean difference) used in the synthesis or presentation of results.	2.3
Synthesis methods	13a	Describe the processes used to decide which studies were eligible for each synthesis (e.g. tabulating the study intervention characteristics and comparing against the planned groups for each synthesis (item #5)).	2.3
	13b	Describe any methods required to prepare the data for presentation or synthesis, such as handling of missing summary statistics, or data conversions.	2.3
	13c	Describe any methods used to tabulate or visually display results of individual studies and syntheses.	2.3
	13d	Describe any methods used to synthesize results and provide a rationale for the choice(s). If meta-analysis was performed, describe the model(s), method(s) to identify the presence and extent of statistical heterogeneity, and software package(s) used.	2.3
	13e	Describe any methods used to explore possible causes of heterogeneity among study results (e.g. subgroup analysis, meta-regression).	2.3
	13f	Describe any sensitivity analyses conducted to assess robustness of the synthesized results.	2.3
Reporting bias assessment	14	Describe any methods used to assess risk of bias due to missing results in a synthesis (arising from reporting biases).	2.3
Certainty assessment	15	Describe any methods used to assess certainty (or confidence) in the body of evidence for an outcome.	2.3
RESULTS			
Study selection	16a	Describe the results of the search and selection process, from the number of records identified in the search to the number of studies included in the review, ideally using a flow diagram.	3
	16b	Cite studies that might appear to meet the inclusion criteria, but which were excluded, and explain why they were excluded.	3; PRISMA flow chart
Study characteristics	17	Cite each included study and present its characteristics.	3.1; table 1
Risk of bias in studies	18	Present assessments of risk of bias for each included study.	3.2
Results of individual studies	19	For all outcomes, present, for each study: (a) summary statistics for each group (where appropriate) and (b) an effect estimate and its precision (e.g. confidence/credible interval), ideally using structured tables or plots.	3.3; 3.4; Table 2 & 3
Results of syntheses	20a	For each synthesis, briefly summarise the characteristics and risk of bias among contributing studies.	3.3; 3.4
	20b	Present results of all statistical syntheses conducted. If meta-analysis was done, present for each the summary estimate and its precision (e.g. confidence/credible interval) and measures of statistical heterogeneity. If comparing groups, describe the direction of the effect.	3.3; 3.4; Table 2 & 3
	20c	Present results of all investigations of possible causes of heterogeneity among study results.	/
	20d	Present results of all sensitivity analyses conducted to assess the robustness of the synthesized results.	Table 2 & 3
Reporting biases	21	Present assessments of risk of bias due to missing results (arising from reporting biases) for each synthesis assessed.	3.2; supplementary materials
Certainty of evidence	22	Present assessments of certainty (or confidence) in the body of evidence for each outcome assessed.	3.3; 3.4
DISCUSSION			
Discussion	23a	Provide a general interpretation of the results in the context of other evidence.	4
	23b	Discuss any limitations of the evidence included in the review.	4
	23c	Discuss any limitations of the review processes used.	4
	23d	Discuss implications of the results for practice, policy, and future research.	4
OTHER INFORMATION			
Registration and protocol	24a	Provide registration information for the review, including register name and registration number, or state that the review was not registered.	2
	24b	Indicate where the review protocol can be accessed, or state that a protocol was not prepared.	2
	24c	Describe and explain any amendments to information provided at registration or in the protocol.	2.1
Support	25	Describe sources of financial or non-financial support for the review, and the role of the funders or sponsors in the review.	acknowledgements
Competing interests	26	Declare any competing interests of review authors.	acknowledgements
Availability of data, code and other materials	27	Report which of the following are publicly available and where they can be found: template data collection forms; data extracted from included studies; data used for all analyses; analytic code; any other materials used in the review.	acknowledgements

Figure S1. Forest plots of intermediate dose anticoagulation vs standard dose thromboprophylaxis for the outcomes (1) all-cause mortality at 30 days, (2) all-cause mortality at 90 days, (3) any thrombotic event or death, (4) any thrombotic event at 30 days and (5) major bleeding at 30 days. Risk of Bias assessment graded as +, no concern in according domain; ?, some concern in according domain domain

Comparison 2: JCM - Anticoagulation: intermediate dose anticoagulation versus standard thromboprophylaxis (low dose), Outcome 1: All-cause mortality (30 days)

Study or Subgroup	intermediate dose anticoagulation Events	intermediate dose anticoagulation Total	standard thromboprophylaxis (low dose) Events	standard thromboprophylaxis (low dose) Total	Weight	Risk Ratio M-H, Random, 95% CI
**14.1.1 Mixed moderate to severe disease (WHO 4 to 9)**						
Perepu 2021	13	87	18	86	17.4%	0.71 [0.37 , 1.37]
Sadeghipour 2021, INSPIRATION	124	296	117	294	82.6%	1.05 [0.87 , 1.28]
**Subtotal (95% CI)**		**383**		**380**	**100.0%**	**0.98 [0.74 , 1.32]**
Total events:	137		135			
Heterogeneity: Tau² = 0.02; Chi² = 1.28, df = 1 (P = 0.26); I² = 22%						
Test for overall effect: Z = 0.11 (P = 0.91)						
**Total (95% CI)**		**383**		**380**	**100.0%**	**0.98 [0.74 , 1.32]**
Total events:	137		135			
Heterogeneity: Tau² = 0.02; Chi² = 1.28, df = 1 (P = 0.26); I² = 22%						
Test for overall effect: Z = 0.11 (P = 0.91)						
Test for subgroup differences: Not applicable						

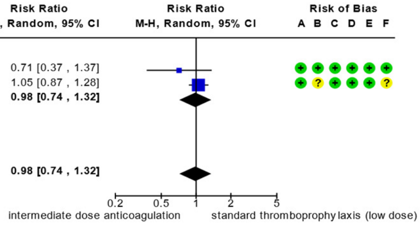

Risk of bias legend
(A) Bias arising from the randomization process
(B) Bias due to deviations from intended interventions
(C) Bias due to missing outcome data
(D) Bias in measurement of the outcome
(E) Bias in selection of the reported result
(F) Overall bias

Comparison 2: JCM - Anticoagulation: intermediate dose anticoagulation versus standard thromboprophylaxis (low dose), Outcome 2: All-cause mortality (90 days)

Study or Subgroup	intermediate dose anticoagulation Events	intermediate dose anticoagulation Total	standard thromboprophylaxis (low dose) Events	standard thromboprophylaxis (low dose) Total	Weight	Risk Ratio M-H, Random, 95% CI
**14.2.1 Mixed moderate to severe disease (WHO 4 to 9)**						
Sadeghipour 2021, INSPIRATION (1)	132	296	123	294	100.0%	1.07 [0.89 , 1.28]
**Subtotal (95% CI)**		**296**		**294**	**100.0%**	**1.07 [0.89 , 1.28]**
Total events:	132		123			
Heterogeneity: Not applicable						
Test for overall effect: Z = 0.68 (P = 0.50)						
**Total (95% CI)**		**296**		**294**	**100.0%**	**1.07 [0.89 , 1.28]**
Total events:	132		123			
Heterogeneity: Not applicable						
Test for overall effect: Z = 0.68 (P = 0.50)						
Test for subgroup differences: Not applicable						

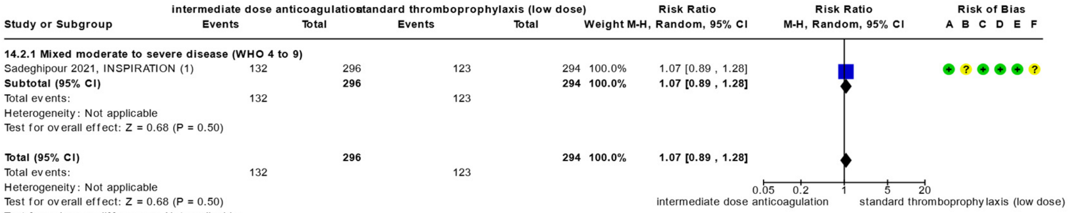

Footnotes
(1) Data from Bikdeli-2021

Risk of bias legend
(A) Bias arising from the randomization process
(B) Bias due to deviations from intended interventions
(C) Bias due to missing outcome data
(D) Bias in measurement of the outcome
(E) Bias in selection of the reported result
(F) Overall bias

Comparison 2: JCM - Anticoagulation: intermediate dose anticoagulation versus standard thromboprophylaxis (low dose), Outcome 3: Any thrombotic event (venous or arterial thrombosis) or death (within 30 days)

Study or Subgroup	intermediate dose anticoagulation Events	intermediate dose anticoagulation Total	standard thromboprophylaxis (low dose) Events	standard thromboprophylaxis (low dose) Total	Weight	Risk Ratio M-H, Random, 95% CI
**14.3.1 Mixed moderate to severe disease (WHO 4 to 9)**						
Sadeghipour 2021, INSPIRATION	131	296	126	294	100.0%	1.03 [0.86 , 1.24]
**Subtotal (95% CI)**		**296**		**294**	**100.0%**	**1.03 [0.86 , 1.24]**
Total events:	131		126			
Heterogeneity: Not applicable						
Test for overall effect: Z = 0.34 (P = 0.73)						
**Total (95% CI)**		**296**		**294**	**100.0%**	**1.03 [0.86 , 1.24]**
Total events:	131		126			
Heterogeneity: Not applicable						
Test for overall effect: Z = 0.34 (P = 0.73)						
Test for subgroup differences: Not applicable						

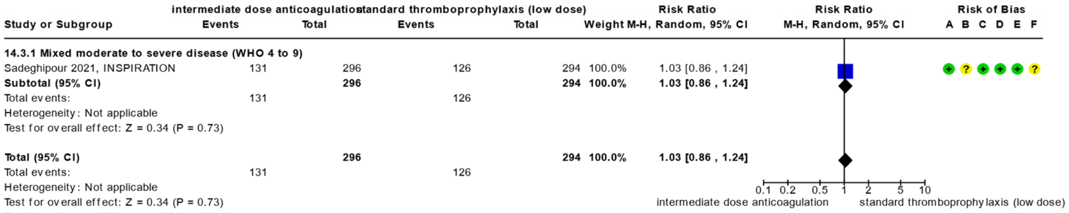

Risk of bias legend
(A) Bias arising from the randomization process
(B) Bias due to deviations from intended interventions
(C) Bias due to missing outcome data
(D) Bias in measurement of the outcome
(E) Bias in selection of the reported result
(F) Overall bias

Comparison 2: JCM - Anticoagulation: intermediate dose anticoagulation versus standard thromboprophylaxis (low dose), Outcome 4: Any venous thrombotic event (30 days)

Study or Subgroup	intermediate dose anticoagulation Events	Total	standard thromboprophylaxis (low dose) Events	Total	Weight	Risk Ratio M-H, Random, 95% CI
**14.4.1 Mixed moderate to severe disease (WHO 4 to 9)**						
Perepu 2021	7	87	6	86	41.6%	1.15 [0.40 , 3.29]
Sadeghipour 2021, INSPIRATION	9	296	10	294	58.4%	0.89 [0.37 , 2.17]
**Subtotal (95% CI)**		**383**		**380**	**100.0%**	**0.99 [0.51 , 1.96]**
Total events:	16		16			
Heterogeneity: Tau² = 0.00; Chi² = 0.13, df = 1 (P = 0.72); I² = 0%						
Test for overall effect: Z = 0.02 (P = 0.99)						
**Total (95% CI)**		**383**		**380**	**100.0%**	**0.99 [0.51 , 1.96]**
Total events:	16		16			
Heterogeneity: Tau² = 0.00; Chi² = 0.13, df = 1 (P = 0.72); I² = 0%						
Test for overall effect: Z = 0.02 (P = 0.99)						
Test for subgroup differences: Not applicable						

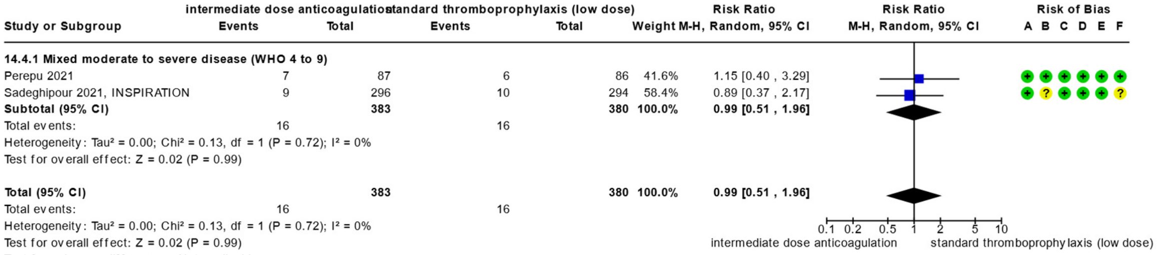

Risk of bias legend
(A) Bias arising from the randomization process
(B) Bias due to deviations from intended interventions
(C) Bias due to missing outcome data
(D) Bias in measurement of the outcome
(E) Bias in selection of the reported result
(F) Overall bias

Comparison 2: JCM - Anticoagulation: intermediate dose anticoagulation versus standard thromboprophylaxis (low dose), Outcome 5: Major bleeding up to 30 days

Study or Subgroup	intermediate dose anticoagulation Events	Total	standard thromboprophylaxis (low dose/intermediate dose) Events	Total	Weight	Risk Ratio M-H, Random, 95% CI
**14.5.1 Mixed moderate to severe disease (WHO 4 to 9)**						
Perepu 2021	2	87	2	86	28.3%	0.99 [0.14 , 6.86]
Sadeghipour 2021, INSPIRATION	7	296	4	294	71.7%	1.74 [0.51 , 5.87]
**Subtotal (95% CI)**		**383**		**380**	**100.0%**	**1.48 [0.53 , 4.15]**
Total events:	9		6			
Heterogeneity: Tau² = 0.00; Chi² = 0.23, df = 1 (P = 0.63); I² = 0%						
Test for overall effect: Z = 0.75 (P = 0.46)						
**Total (95% CI)**		**383**		**380**	**100.0%**	**1.48 [0.53 , 4.15]**
Total events:	9		6			
Heterogeneity: Tau² = 0.00; Chi² = 0.23, df = 1 (P = 0.63); I² = 0%						
Test for overall effect: Z = 0.75 (P = 0.46)						
Test for subgroup differences: Not applicable						

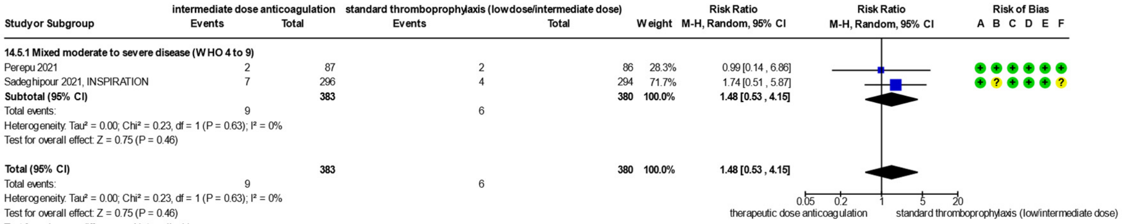

Risk of bias legend
(A) Bias arising from the randomization process
(B) Bias due to deviations from intended interventions
(C) Bias due to missing outcome data
(D) Bias in measurement of the outcome
(E) Bias in selection of the reported result
(F) Overall bias

Figure S2. Forest plots according to pre-specified subgroups (moderately and severely diseased population) of therapeutic dose anticoagulation vs standard dose thromboprophylaxis for the outcomes (1) all-cause mortality at 28-30 days, (2) all-cause mortality in hospital, (3) worsening of clinical status: progression to intubation aor death at 28 days, (4) clinical worsening: progression to any mechanical ventilation or death at 28 days, (5) improvement of clnical status: participants discharged alive, (6) improvement of clinical status: survival until hospital discharge without receiving organ support, (7) any thrombotic event or death at 28 to 30 days, (8) any thrombotic event at 28-30 days and (9) major bleeding (ISTH) during treatment/up to 30 days. Risk of Bias assessment graded as +, no concern in according domain; ? some concern in according domain

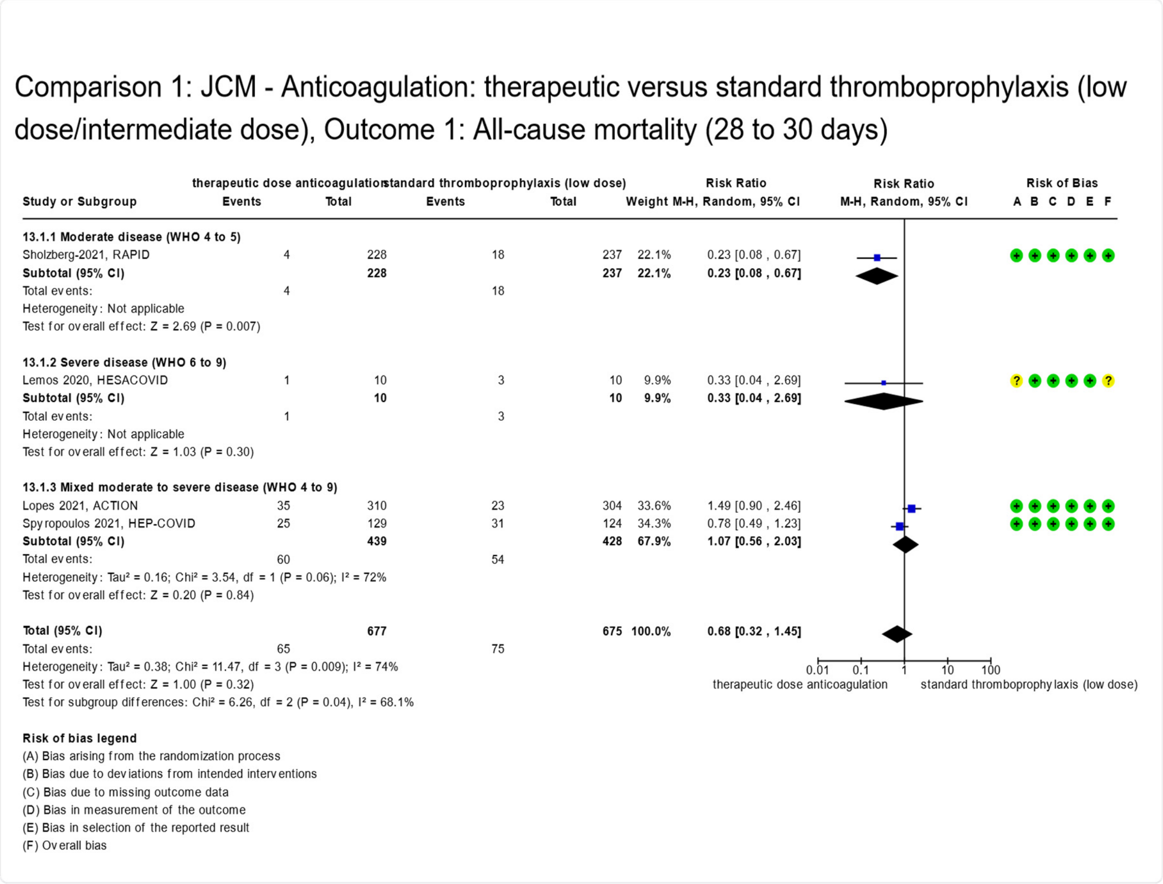

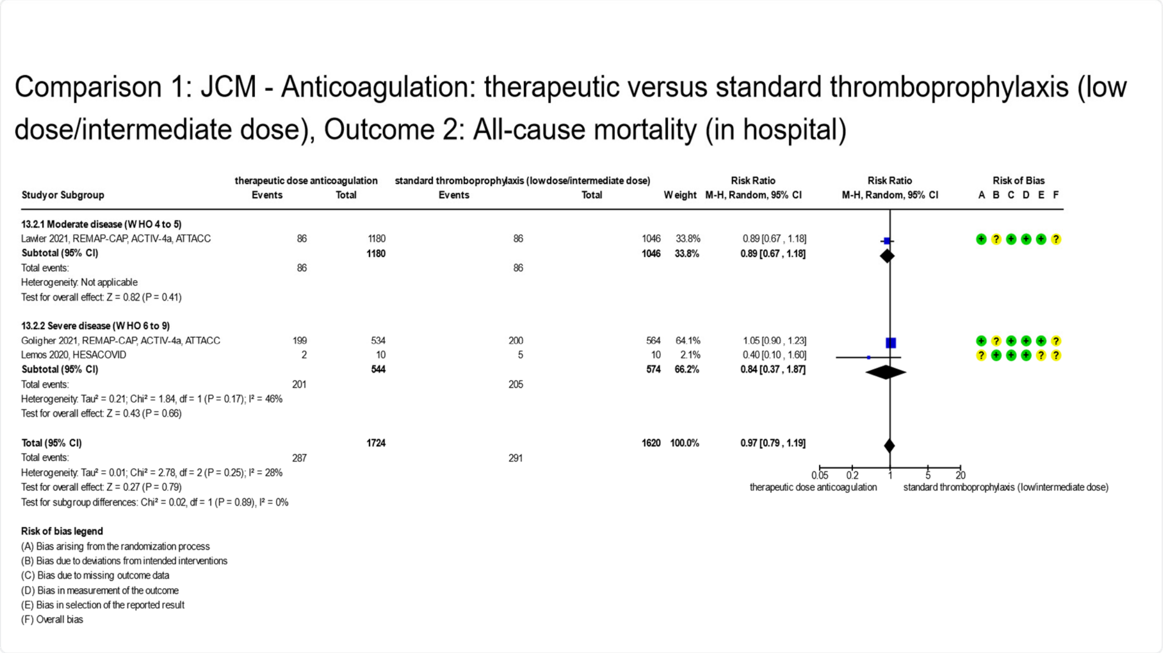

Comparison 1: JCM - Anticoagulation: therapeutic versus standard thromboprophylaxis (low dose/intermediate dose), Outcome 3: Worsening of clinical status: Progression to intubation or death (28 days)

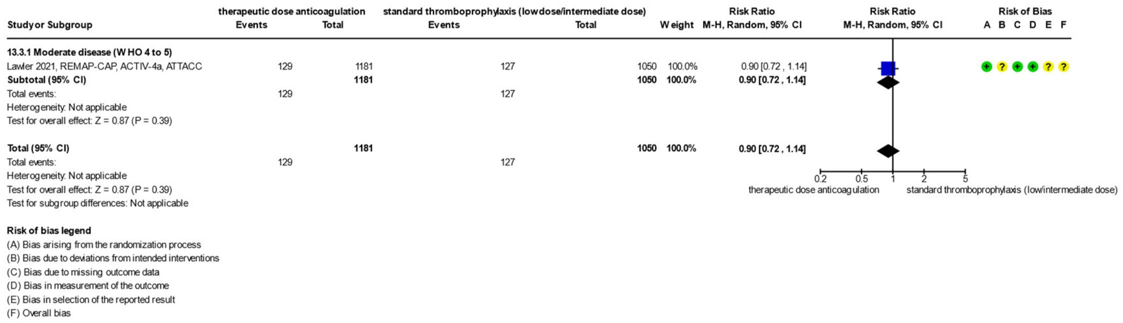

Comparison 1: JCM - Anticoagulation: therapeutic versus standard thromboprophylaxis (low dose/intermediate dose), Outcome 4: Worsening of clinical status: Progression to any mechanical ventilation or death (28 days)

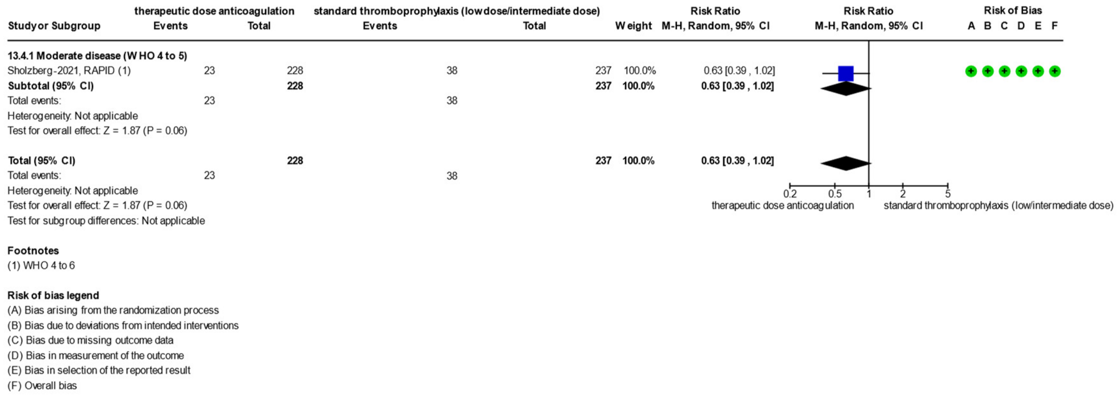

Comparison 1: JCM - Anticoagulation: therapeutic versus standard thromboprophylaxis (low dose/intermediate dose), Outcome 5: Improvement of clinical status: Participants discharged alive without clinical deterioration or death

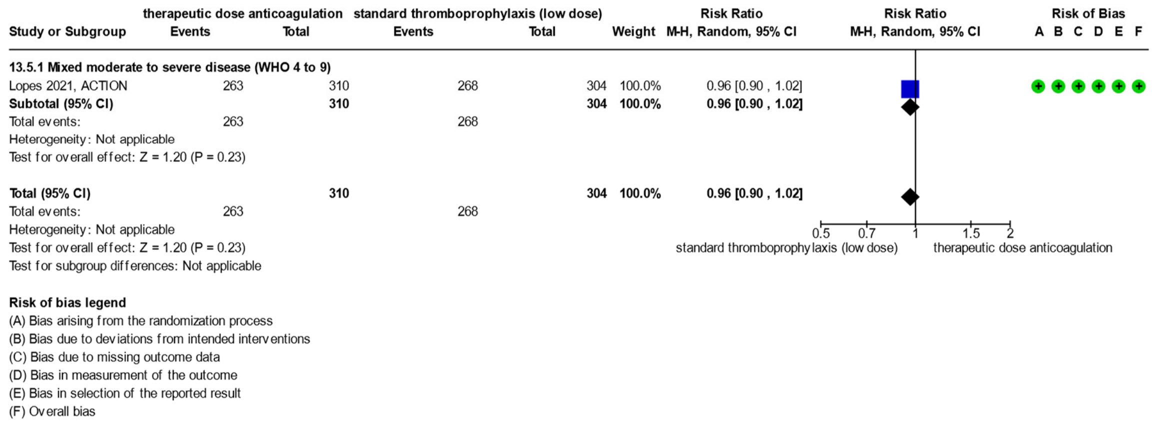

Comparison 1: JCM - Anticoagulation: therapeutic versus standard thromboprophylaxis (low dose/intermediate dose), Outcome 6: Improvement of clinical status: Survival until hospital discharge without receiving organ support

Study or Subgroup	therapeutic dose anticoagulation Events	Total	standard thromboprophylaxis (low dose/intermediate dose) Events	Total	Weight	Risk Ratio M-H, Random, 95% CI	A	B	C	D	E	F
**13.6.1 Moderate disease (WHO 4 to 5)**												
Lawler 2021, REMAP-CAP, ACTIV-4a, ATTACC	939	1171	801	1048	100.0%	1.05 [1.00 , 1.10]	+	?	+	+	+	?
**Subtotal (95% CI)**		**1171**		**1048**	**100.0%**	**1.05 [1.00 , 1.10]**						
Total events:	939		801									
Heterogeneity: Not applicable												
Test for overall effect: Z = 2.13 (P = 0.03)												
**Total (95% CI)**		**1171**		**1048**	**100.0%**	**1.05 [1.00 , 1.10]**						
Total events:	939		801									
Heterogeneity: Not applicable												
Test for overall effect: Z = 2.13 (P = 0.03)												
Test for subgroup differences: Not applicable												

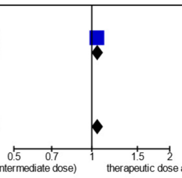

Risk of bias legend
(A) Bias arising from the randomization process
(B) Bias due to deviations from intended interventions
(C) Bias due to missing outcome data
(D) Bias in measurement of the outcome
(E) Bias in selection of the reported result
(F) Overall bias

Comparison 1: JCM - Anticoagulation: therapeutic versus standard thromboprophylaxis (low dose/intermediate dose), Outcome 7: Any thrombotic event or death (28 to 30 days)

Study or Subgroup	therapeutic dose anticoagulation Events	Total	standard thromboprophylaxis (low dose/intermediate dose) Events	Total	Weight	Risk Ratio M-H, Random, 95% CI	A	B	C	D	E	F
**13.7.1 Moderate disease (WHO 4 to 5)**												
Lawler 2021, REMAP-CAP, ACTIV-4a, ATTACC	96	1180	108	1046	24.1%	0.79 [0.61 , 1.02]	+	?	+	+	+	?
Spyropoulos 2021, HEP-COVID	14	84	31	86	10.0%	0.46 [0.27 , 0.81]	+	+	+	+	+	+
**Subtotal (95% CI)**		**1264**		**1132**	**34.1%**	**0.64 [0.38 , 1.07]**						
Total events:	110		139									
Heterogeneity: Tau² = 0.09; Chi² = 2.90, df = 1 (P = 0.09); I² = 66%												
Test for overall effect: Z = 1.72 (P = 0.09)												
**13.7.2 Severe disease (WHO 6 to 9)**												
Goligher 2021, REMAP-CAP, ACTIV-4a, ATTACC	217	531	232	560	33.9%	0.99 [0.86 , 1.14]	+	?	+	+	+	?
Spyropoulos 2021, HEP-COVID (1)	23	45	21	38	15.5%	0.92 [0.62 , 1.39]	+	+	+	+	+	+
**Subtotal (95% CI)**		**576**		**598**	**49.3%**	**0.98 [0.86 , 1.12]**						
Total events:	240		253									
Heterogeneity: Tau² = 0.00; Chi² = 0.09, df = 1 (P = 0.77); I² = 0%												
Test for overall effect: Z = 0.30 (P = 0.76)												
**13.7.3 Mixed moderate to severe disease (WHO 4 to 9)**												
Lopes 2021, ACTION (2)	46	310	44	304	16.6%	1.03 [0.70 , 1.50]	+	+	+	?	+	?
**Subtotal (95% CI)**		**310**		**304**	**16.6%**	**1.03 [0.70 , 1.50]**						
Total events:	46		44									
Heterogeneity: Not applicable												
Test for overall effect: Z = 0.13 (P = 0.90)												
**Total (95% CI)**		**2150**		**2034**	**100.0%**	**0.86 [0.71 , 1.06]**						
Total events:	396		436									
Heterogeneity: Tau² = 0.03; Chi² = 8.61, df = 4 (P = 0.07); I² = 54%												
Test for overall effect: Z = 1.43 (P = 0.15)												
Test for subgroup differences: Chi² = 2.63, df = 2 (P = 0.27), I² = 24.0%												

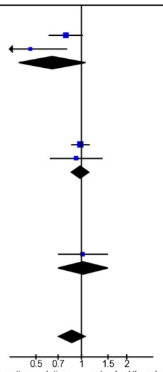

Footnotes
(1) WHO 6-7
(2) 85% WHO 4-5

Risk of bias legend
(A) Bias arising from the randomization process
(B) Bias due to deviations from intended interventions
(C) Bias due to missing outcome data
(D) Bias in measurement of the outcome
(E) Bias in selection of the reported result
(F) Overall bias

Comparison 1: JCM - Anticoagulation: therapeutic versus standard thromboprophylaxis (low dose/intermediate dose), Outcome 8: Any thrombotic event (28 to 30 days)

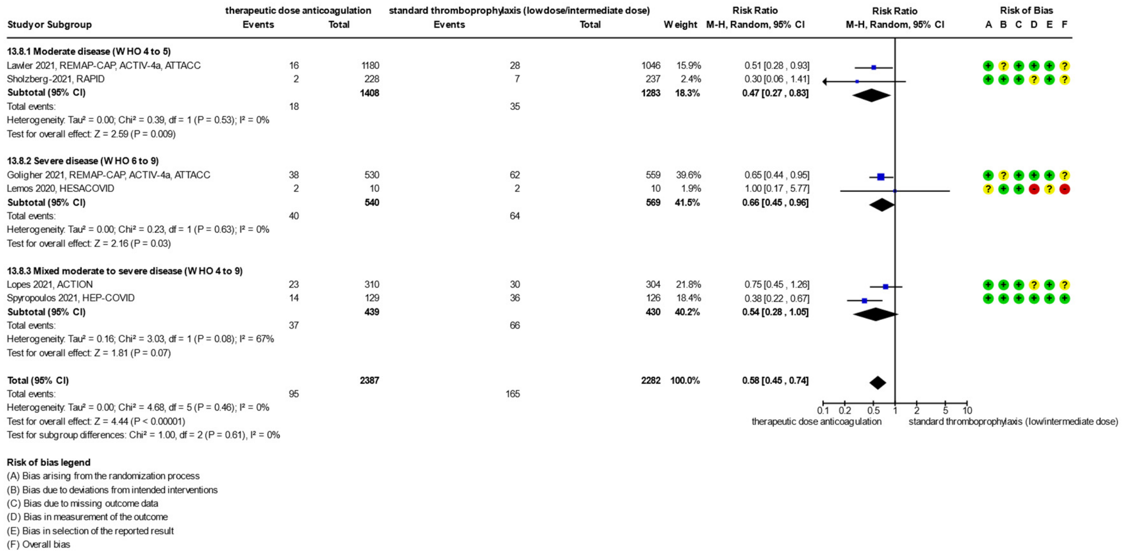

Comparison 1: JCM - Anticoagulation: therapeutic versus standard thromboprophylaxis (low dose/intermediate dose), Outcome 9: Major bleeding (ISTH) during treatment/up to 30 days

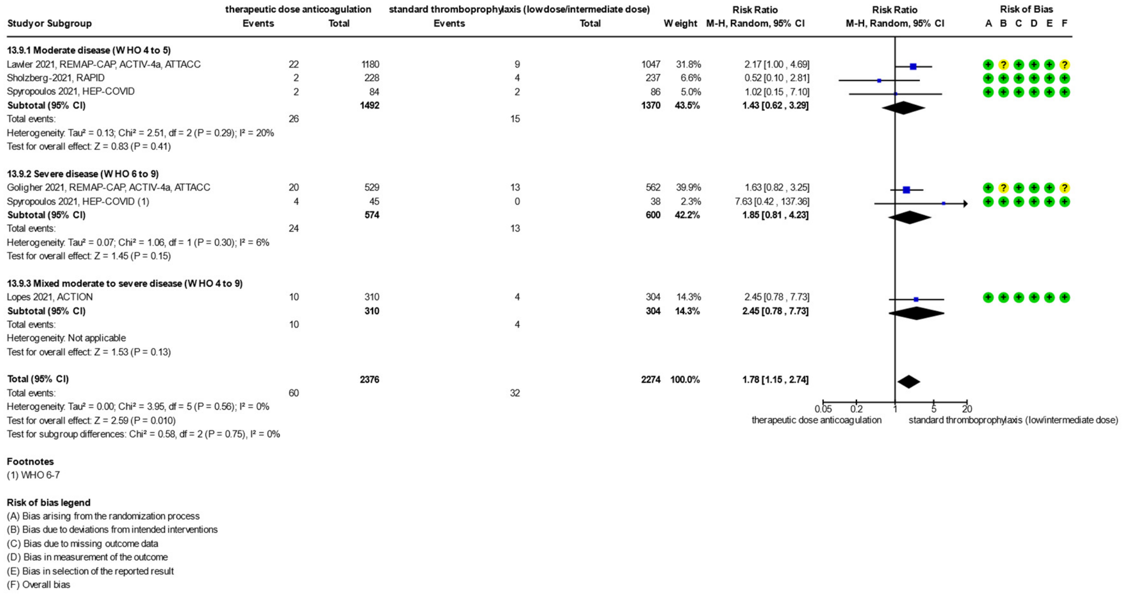

Search strategy

All searches were conducted on 24 September 2021 and deduplicated using EndNote X8.

Cochrane COVID-19 Study Register (https://covid-19.cochrane.org)

Search string:

anticoagula* OR antithromb* OR "Thrombin Inhibitor" OR "Thrombin Inhibitors" OR Dabigatran OR Pradaxa OR Argatroban OR Novastan OR Acova OR Lepirudin OR Refludan OR Desirudin OR Iprivask OR Revasc OR desulfatohirudin* OR "recombinant HV1 hirudin" OR Bivalirudin OR Hirulog* OR Angiomax OR Angiox OR "Xa inhibitor" OR "Xa inhibitors" OR Xaban* OR Rivaroxaban OR Xarelto OR Apixaban OR Eliquis OR Edoxaban OR Lixiana OR Savaysa OR coumar* OR cumar* OR kumar* OR Benzopyrone* OR Benzopyran* OR Hydroxycinnamic OR "Tonka bean camphor" OR "Vitamin K antagonist" OR "Vitamin K antagonists" OR phenprocoumon* OR henylpropylhydroxycumarin* OR Falithrom OR Fencumar OR Fenprocoumon* OR Liquamar OR Marcoumar OR Marcumar OR Phenprogramma OR Warfarin* OR Warfarat OR Aldocumar OR Warfant OR Brumolin OR Coumefene OR Dethmor OR Dethnel OR Kypfarin OR Marevan OR Panwarfin OR Prothromadin OR Tedicumar OR Zoocoumarin OR Heparin* OR Liquaemin OR Adomiparin OR Ardeparin OR Arteven OR Bemiparin* OR Certoparin OR Clexane OR Klexane OR Clivarin* OR Dalteparin OR Eparina OR Fluxum OR "Fragmin A" OR "Fragmin B" OR Fraxiparin OR Hepathrom OR "Lipo-hepin" OR Liquemin OR Multiparin OR Nadroparin* OR Novoheparin OR Octaparin OR Pabyrin OR Parnaparin* OR Parvoparin OR Pularin OR Reviparin OR Sandoparin OR Semuloparin OR Subeparin OR Sublingula OR Thromboliquine OR Tinzaparin* OR Triofiban OR Vetren OR "Vitrum AB" OR UFH OR LMWH OR Alphaparin* OR "Mono-Embolex" OR Enoxaparin* OR Lovenox OR Danaparoid OR Danaproid OR Orgaran OR Lomoparan OR Fondaparinux OR Penta OR Quixidar OR Arixtra OR sulodexid* OR Aterina OR Luzone OR "glucuronyl glucosamine glycan sulfate" OR "glucuronyl glucosaminoglycan sulfate" OR Dociparastat

Study characteristics:

1) "Intervention assignment": “Randomised” OR “quasi-randomised” or “unclear” OR

2) "Study type": "Interventional" AND "Study design": "Parallel/Crossover"

3) "Study type": "Interventional" AND "Study design": "Unclear"

4) "Study type": "Adaptive/Platform"

= 245 studies (431 references)

Clarivate Web of Science Core Collection (Advanced search)

#1

TI=(anticoagula* OR antithromb* OR "Thrombin Inhibitor*" OR Dabigatran OR Pradaxa OR Argatroban OR Novastan OR Acova OR Lepirudin OR Refludan OR Desirudin OR Iprivask OR Revasc OR desulfatohirudin* OR "recombinant HV1 hirudin" OR Bivalirudin OR Hirulog* OR Angiomax OR Angiox OR "Xa inhibitor*" OR Xaban* OR Rivaroxaban OR Xarelto OR Apixaban OR Eliquis OR Edoxaban OR Lixiana OR Savaysa OR coumar* OR cumar* OR kumar* OR Benzopyrone* OR Benzopyran* OR Hydroxycinnamic OR "Tonka bean camphor" OR "Vitamin K antagonist" OR "Vitamin K antagonists" OR phenprocoumon* OR henylpropylhydroxycumarin* OR Falithrom OR Fencumar OR Fenprocoumon* OR Liquamar OR Marcoumar OR Marcumar OR Phenprogramma OR Warfarin* OR Warfarat OR Aldocumar OR Warfant OR Brumolin OR Coumefene OR Dethmor OR Dethnel OR Kypfarin OR Marevan OR Panwarfin OR Prothromadin OR Tedicumar OR Zoocoumarin OR Heparin* OR Liquaemin OR Adomiparin OR Ardeparin OR Arteven OR Bemiparin* OR Certoparin OR Clexane OR Klexane OR Clivarin* OR Dalteparin OR Eparina OR Fluxum OR "Fragmin A" OR "Fragmin B" OR Fraxiparin OR Hepathrom OR "Lipo-hepin" OR Liquemin OR Multiparin

OR Nadroparin* OR Novoheparin OR Octaparin OR Pabyrin OR Parnaparin* OR Parvoparin OR Pularin OR Reviparin OR Sandoparin OR Semuloparin OR Subeparin OR Sublingula OR Thromboliquine OR Tinzaparin* OR Triofiban OR Vetren OR "Vitrum AB" OR UFH OR LMWH OR Alphaparin* OR "Mono-Embolex" OR Enoxaparin* OR Lovenox OR Danaparoid OR Danaproid OR Orgaran OR Lomoparan OR Fondaparinux OR Penta OR Quixidar OR Arixtra OR sulodexid* OR Aterina OR Luzone OR "glucuronyl glucosamine glycan sulfate" OR "glucuronyl glucosaminoglycan sulfate" OR Dociparastat) OR AB=(anticoagula* OR antithromb* OR "Thrombin Inhibitor*" OR Dabigatran OR Pradaxa OR Argatroban OR Novastan OR Acova OR Lepirudin OR Refludan OR Desirudin OR Iprivask OR Revasc OR desulfatohirudin* OR "recombinant HV1 hirudin" OR Bivalirudin OR Hirulog* OR Angiomax OR Angiox OR "Xa inhibitor*" OR Xaban* OR Rivaroxaban OR Xarelto OR Apixaban OR Eliquis OR Edoxaban OR Lixiana OR Savaysa OR coumar* OR cumar* OR kumar* OR Benzopyrone* OR Benzopyran* OR Hydroxycinnamic OR "Tonka bean camphor" OR "Vitamin K antagonist" OR "Vitamin K antagonists" OR phenprocoumon* OR henylpropylhydroxycumarin* OR Falithrom OR Fencumar OR Fenprocoumon* OR Liquamar OR Marcoumar OR Marcumar OR Phenprogramma OR Warfarin* OR Warfarat OR Aldocumar OR Warfant OR Brumolin OR Coumefene OR Dethmor OR Dethnel OR Kypfarin OR Marevan OR Panwarfin OR Prothromadin OR Tedicumar OR Zoocoumarin OR Heparin* OR Liquaemin OR Adomiparin OR Ardeparin OR Arteven OR Bemiparin* OR Certoparin OR Clexane OR Klexane OR Clivarin* OR Dalteparin OR Eparina OR Fluxum OR "Fragmin A" OR "Fragmin B" OR Fraxiparin OR Hepathrom OR "Lipo-hepin" OR Liquemin OR Multiparin OR Nadroparin* OR Novoheparin OR Octaparin OR Pabyrin OR Parnaparin* OR Parvoparin OR Pularin OR Reviparin OR Sandoparin OR Semuloparin OR Subeparin OR Sublingula OR Thromboliquine OR Tinzaparin* OR Triofiban OR Vetren OR "Vitrum AB" OR UFH OR LMWH OR Alphaparin* OR "Mono-Embolex" OR Enoxaparin* OR Lovenox OR Danaparoid OR Danaproid OR Orgaran OR Lomoparan OR Fondaparinux OR Penta OR Quixidar OR Arixtra OR sulodexid* OR Aterina OR Luzone OR "glucuronyl glucosamine glycan sulfate" OR "glucuronyl glucosaminoglycan sulfate" OR Dociparastat)

#2

TI=(COVID OR COVID19 OR "SARS-CoV-2" OR "SARS-CoV2" OR SARSCoV2 OR "SARSCoV-2" OR "SARS coronavirus 2" OR "2019 nCoV" OR "2019nCoV" OR "2019-novel CoV" OR "nCov 2019" OR "nCov 19" OR "severe acute respiratory syndrome coronavirus 2" OR "novel coronavirus disease" OR "novel corona virus disease" OR "corona virus disease 2019" OR "coronavirus disease 2019" OR "novel coronavirus pneumonia" OR "novel corona virus pneumonia" OR "severe acute respiratory syndrome coronavirus 2") OR AB=(COVID OR COVID19 OR "SARS-CoV-2" OR "SARS-CoV2" OR SARSCoV2 OR "SARSCoV-2" OR "SARS coronavirus 2" OR "2019 nCoV" OR "2019nCoV" OR "2019-novel CoV" OR "nCov 2019" OR "nCov 19" OR "severe acute respiratory syndrome coronavirus 2" OR "novel coronavirus disease" OR "novel corona virus disease" OR "corona virus disease 2019" OR "coronavirus disease 2019" OR "novel coronavirus pneumonia" OR "novel corona virus pneumonia" OR "severe acute respiratory syndrome coronavirus 2")

#3

TI=(random* OR placebo OR trial OR groups OR "phase 3" OR "phase3" OR p3 OR "pIII") OR AB=(random* OR placebo OR trial OR groups OR "phase 3" OR "phase3" OR p3 OR "pIII")

#4

#1 AND #2 AND #3

Indexes=SCI-EXPANDED, ESCI

= 471 references

WHO COVID-19 Global literature on coronavirus disease

(https://search.bvsalud.org/global-literature-on-novel-coronavirus-2019-ncov)

Title, abstract, subject:
(anticoagula* OR antithromb* OR "Thrombin Inhibitor" OR "Thrombin Inhibitors" OR Dabigatran OR Pradaxa OR Argatroban OR Novastan OR Acova OR Lepirudin OR Refludan OR Desirudin OR Iprivask OR Revasc OR desulfatohirudin* OR "recombinant HV1 hirudin" OR Bivalirudin OR Hirulog* OR Angiomax OR Angiox OR "Xa inhibitor" OR "Xa inhibitors" OR Xaban* OR Rivaroxaban OR Xarelto OR Apixaban OR Eliquis OR Edoxaban OR Lixiana OR Savaysa OR coumar* OR cumar* OR kumar* OR Benzopyrone* OR Benzopyran* OR Hydroxycinnamic OR "Tonka bean camphor" OR "Vitamin K antagonist" OR "Vitamin K antagonists" OR phenprocoumon* OR henylpropylhydroxycumarin* OR Falithrom OR Fencumar OR Fenprocoumon* OR Liquamar OR Marcoumar OR Marcumar OR Phenprogramma OR Warfarin* OR Warfarat OR Aldocumar OR Warfant OR Brumolin OR Coumefene OR Dethmor OR Dethnel OR Kypfarin OR Marevan OR Panwarfin OR Prothromadin OR Tedicumar OR Zoocoumarin OR Heparin* OR Liquaemin OR Adomiparin OR Ardeparin OR Arteven OR Bemiparin* OR Certoparin OR Clexane OR Klexane OR Clivarin* OR Dalteparin OR Eparina OR Fluxum OR "Fragmin A" OR "Fragmin B" OR Fraxiparin OR Hepathrom OR "Lipo-hepin" OR Liquemin OR Multiparin OR Nadroparin* OR Novoheparin OR Octaparin OR Pabyrin OR Parnaparin* OR Parvoparin OR Pularin OR Reviparin OR Sandoparin OR Semuloparin OR Subeparin OR Sublingula OR Thromboliquine OR Tinzaparin* OR Triofiban OR Vetren OR "Vitrum AB" OR UFH OR LMWH OR Alphaparin* OR "Mono-Embolex" OR Enoxaparin* OR Lovenox OR Danaparoid OR Danaproid OR Orgaran OR Lomoparan OR Fondaparinux OR Penta OR Quixidar OR Arixtra OR sulodexid* OR Aterina OR Luzone OR "glucuronyl glucosamine glycan sulfate" OR "glucuronyl glucosaminoglycan sulfate" OR Dociparastat) AND (random* OR placebo OR trial OR groups OR "phase 3" or "phase3" or p3 or "pIII")

→ excluding databases: MEDLINE, ICTRP, EMBASE, Scopus, PubMed, PMC, Web of Science
= 250 references

ResearchSquare

(https://www.researchsquare.com)

Article type: Research Article
Abstract:
- anticoagulant = 35
- antithrombotic = 2
- thrombin = 2
= selected on website: 1 relevant reference
